# *CsMYB60* is a key regulator of flavonols and proanthocyanidans that determine the colour of fruit spines in cucumber

**DOI:** 10.1093/jxb/ery336

**Published:** 2018-09-26

**Authors:** Mengyu Liu, Cunjia Zhang, Lixin Duan, Qianqian Luan, Jialin Li, Aigang Yang, Xiaoquan Qi, Zhonghai Ren

**Affiliations:** 1 State Key Laboratory of Corp Biology, Shandong Collaborative Innovation Center of Fruit & Vegetable Quality and Efficient Production, Key Laboratory of Biology and Genetic Improvement of Horticultural Crops in Huang-Huai Region, Ministry of Agriculture, College of Horticultural Science and Engineering, Shandong Agricultural University, Tai’an, Shandong, China; 2 The Key Laboratory of Plant Molecular Physiology, Institute of Botany, Chinese Academy of Sciences, Nanxincun, Xiangshan, Beijing, China; 3 International Institute for Translational Chinese Medicine, Guangzhou University of Chinese Medicine, Guangzhou, Guangdong, China; 4 State Key Laboratory of Natural and Biomimetic Drugs, Peking University, Beijing, China

**Keywords:** *CsMYB60*, cucumber, epigenetic alteration, flavonols, fruit spine, *Mutator*-like element, proanthocyanidins

## Abstract

Spine colour is an important fruit quality trait that influences the commercial value of cucumber (*Cucumis sativus*). However, little is known about the metabolites and the regulatory mechanisms of their biosynthesis in black spine varieties. In this study, we determined that the pigments of black spines are flavonoids, including flavonols and proanthocyanidins (PAs). We identified *CsMYB60* as the best candidate for the previously identified *B* (*Black spine*) locus. Expression levels of *CsMYB60* and the key genes involved in flavonoid biosynthesis were higher in black-spine inbred lines than that in white-spine lines at different developmental stages. The insertion of a *Mutator*-like element (CsMULE) in the second intron of *CsMYB60* decreased its expression in a white-spine line. Transient overexpression assays indicated that *CsMYB60* is a key regulatory gene and *Cs4CL* is a key structural gene in the pigmentation of black spines. In addition, the DNA methylation level in the *CsMYB60* promoter was much lower in the black-spine line compared with white-spine line. The CsMULE insert may decrease the expression level of *CsMYB60*, causing hindered synthesis of flavonols and PAs in cucumber fruit spines.

## Teaser

Flavonols and proanthocyanidins are the major components that determine the black colouration of cucumber fruit spines and their synthesis is regulated by *CsMYB60*.

## Introduction

Cucumber (*Cucumis sativus*) is an economically important global vegetable crop (Xu *et al*., 2015b). The visual appearance of the fruit is a highly important commercial trait (Chen *et al.*, 2016), and the colour of the fruit spines (specialized trichomes) is one of the characteristics that determines the economic value (Li *et al.*, 2013). Genetic analysis has indicated that the black fruit-spine trait is controlled by a single gene, *B* (*Black* or *brown spine 1*) and it is dominant over the white trait (Walters *et al.*, 2001). Li *et al.* (2013) mapped the *B* gene to a 50-kb region of the short arm of cucumber chromosome 4 (chr.4) based on linkage analysis using 2001 F_2_ plants from a cross between two inbred lines, WI7200 (black-spine, orange mature fruit) and WI7201 (white-spine, cream-coloured mature fruit). In this 50-kb region, a gene encoding a R2R3-MYB transcription factor, later named *CsMYB60* (Zhao *et al.*, 2014), was considered as the best candidate for *B*, because of the presence of a 1-bp deletion in the third intron and a lower expression level of this R2R3-MYB gene in both immature and mature fruit in WI7201 compared with WI7200 (Li *et al.*, 2013). However, more data are needed to confirm that *CsMYB60* is the *B* locus. In addition, the composition of pigments and the mechanisms regulating their biosynthesis in black-spine plants also need to be clarified.

Flavonoids constitute a large family of plant secondary metabolites that are synthesized via the phenylpropanoid pathway, and they can be categorized into chalcones, flavonols, flavanols, anthocyanins, flavones, and proanthocyanidins. They are ubiquitous in the plant kingdom and are beneficial as physiologically active compounds, stress-protecting agents, attractants, and feeding deterrents, as well as playing a general and significant role in plant resistance to pathogens, herbivores, and environmental stress (Treutter, 2006). Moreover, many flavonoids are active principles of medicinal plants and exhibit pharmacological effects (Marles *et al.*, 2003; Yilmaz and Toledo, 2004). Flavonols are present in fruit and vegetables, and they are consumed in considerable amounts in the human diet (Leo and Woodman, 2015). Flavonols have blood pressure-lowering activity (Dauchet *et al.*, 2009). The blood pressure-lowering effects of quercetin were first reported in spontaneously hypertensive rats (SHRs) (Perez-Vizcaino *et al.*, 2001) and subsequently in other animal models of hypertension (Dauchet *et al.*, 2009). In addition to such activity, flavonols modulate tissue antioxidant status by direct scavenging of free radicals (Qin *et al.*, 2008). In some studies, Kaempferol has been shown to improve the survival and function of β-cells and human islets cultured in the presence of high glucose, leading to enhanced insulin secretion (Zhang *et al.*, 2013). Quercetin protects β-cell function and viability, and reduces oxidative damage induced by inflammatory cytokines (Dai *et al.*, 2013). Thus, flavonols may be used as novel anti-diabetic agents (Leo and Woodman, 2015). Proanthocyanidins (PAs; also called condensed tannins) are polymeric end-products of the flavonoid biosynthetic pathway, and their monomeric building blocks, catechin and epicatechin, have cardioprotective (Serafini *et al.*, 2003), anti-cancer (Ahmad *et al.*, 2000), and anti-inflammatory benefits as antioxidants. Moderate levels of PAs in forage crops also improve nitrogen nutrition, reduce urinary nitrogen excretion, and help counter intestinal parasites in the animals that consume them (Aerts *et al.*, 1999).

MYB proteins belong to a large family of transcription factors. Certain MYB proteins are key regulators of flavonoid biosynthesis (Liu *et al.*, 2015). MtPAR is a MYB family transcription factor that positively regulates PA biosynthesis in *Medicago truncatula* (Verdier *et al.*, 2012). Three R2R3-MYB proteins (MYB11, MYB12, and MYB111) activate the early flavonoid biosynthetic genes to regulate flavonol biosynthesis, whilst the later flavonoid biosynthetic genes are activated by the MYB-bHLH-WD40 (MBW) complex to control the production of anthocyanins and PAs (Li, 2014). As a key enzyme, 4-coumarate:CoA ligase (4CL) provides the precursors for flavonoid and lignin biosynthesis (Sun *et al.*, 2015).

Studies in the fields of genomics and epigenetics have recently demonstrated the importance of transposable elements in genome function and evolution (Hirsch and Springer, 2017; Song and Cao, 2017). As transposable elements propagate in plant genomes and attract epigenetic marks, their neo-insertions can lead to the formation of new, heritable epigenetic variants of genes in their vicinity and impact on the host gene regulatory networks (Bucher *et al.*, 2012). For example, a transposon-induced epigenetic change leads to sex determination in melon (Martin *et al.*, 2009). In this case, the transition from male to female flowers in gynoecious lines results from epigenetic changes in the promoter of a transcription factor, CmWIP1, an inhibitor of female flower development. This natural and heritable epigenetic change results from the insertion of a transposon, which is required for the initiation and maintenance of spreading of DNA methylation to the *CmWIP1* promoter.

The transposon *Mutator* was first identified in maize and was later found in other plants, bacteria, fungi, and protozoans (Robertson, 1978; Wang *et al.*, 2016). The typical structure of a *Mutator*-like transposable element (MULE) includes terminated inverted repeats (TIRs; usually 100–500 bp) flanking an internal sequence and one target site duplication (TSD; usually 8–11 bp) flanking each TIR (Wicker *et al.*, 2009; Ferguson *et al.*, 2013). MULEs are especially prevalent in higher plant genomes. For example, the Arabidopsis genome contains at least 200 MULEs (van Leeuwen *et al.*, 2007) and the *Oryza sativa* genome contains more than 10000 copies (Jiang *et al.*, 2004, 2011; Ferguson *et al.*, 2013). MULEs contribute to the evolution of gene innovation and novelty in plant genomes by capturing ectopic genomic sequences and changing DNA methylation (Jiang *et al.*, 2004; Wang *et al.*, 2016). Because MULEs are one of the most active mobile elements in plants, they have been widely employed as a mutagenizing system (Meeley and Briggs, 1995). To date, little is known regarding the functions of MULEs in the evolution of gene innovation and novelty in the genome of cucumber.

In this study, by combining transcriptomic and metabolic profiles, we determined that the pigments of the black fruit spines in cucumber are composed of flavonoids, primarily flavonols and PAs. We revealed the key roles of *CsMYB60*, the best candidate for the *B* gene, and *Cs4CL* in the colouration of black spines, and we found that the decrease in expression of *CsMYB60* in white-spine plants is caused by an insertion of a *Mutator*-like element, which might function in an epigenetic manner.

## Materials and methods

### Plant material and growth conditions

Twenty-two inbred lines of cucumber (*Cucumis sativus* L.; 2*n*=2*x*=14) were used. Plants were grown for two generations each year in a solar greenhouse at the experimental field at Shandong Agricultural University. Standard commercial management was performed during the cultivation period. All spines on fruit from between nodes 8 and 18 of cucumber plants were sampled using a scalpel, and immediately put into liquid nitrogen. The lines RNS8 (white spines) and RNS9 (black spines) were selected for detailed analysis. Unless otherwise stated, the following methods and the results refer to these two lines.

### Imaging of fruit spines

Fruit spines of the lines RNS8 and RNS9 were examined under a dissection microscope (Leica M165 FC) from 2 d before anthesis (DBA) to 12 d after anthesis (DAA), when the fruit were ~35–40 mm long and ~300–350 mm long, respectively.

### NMR experimental procedures

To obtain purified samples for NMR, column chromatography was carried out using a Sephadex LH-20 (GE Healthcare), and HPLC chromatography was performed on an Alltech instrument (426-HPLC pump, Alltech UV-vis-200 detector) equipped with Kromasil semi-preparative (10 μm, ODS, 10 × 250 mm) and YMC-Pack C8 (5 μm, 10 × 250 mm) columns. The purified samples were subjected to NMR analysis. ^1^H and ^13^C NMR and 2D NMR spectra were recorded on an AVANCE 400 FT 400 MHz NMR spectrometer (Bruker) using tetramethylsilane (TMS) as the internal standard. Chemical shifts (δ) are expressed in parts per million (ppm), and coupling constants (J) are reported in Hz. Electrospray ionization (ESI)-MS was conducted using a Bruker APEX IV instrument. Black spines (2.15 g) from RNS9 fruit were homogenized in liquid nitrogen and then extracted with water. After evaporation of the water by rotavapor, the residue (300 mg) was partitioned by water. Samples were then subjected to Sephadex LH-20 column chromatography and eluted with MeOH:H_2_O (1:1) to obtain six fractions (F1–F6). F1 mainly contained sugar (determined by TLC), while F2–F4 contained flavonoid glycoside (detected by UV and 1H NMR). F5 and F6 were mainly low polarity components and only contained trace flavonoid glycosides. F2 was separated by semi-HPLC with CH_3_CN-H_2_O (7%) to obtain purified compound A (5.6 mg), F3 was separated by semi-HPLC with CH_3_CN-H_2_O (8%) to obtain compound B (5.6 mg) and compound C (4.3 mg), and F4 was separated by semi-HPLC with CH_3_CN-H_2_O (10%) to obtain compound D (1.8 mg) and compound E (1.4 mg).

### LC-MS analysis

In order to compare the metabolic profiles of black and white spines, a metabolomics approach was performed. An Agilent 1290 Series UHPLC system was coupled online with a hybrid quadrupole time-of-flight (Q-TOF) mass spectrometer (6540, Agilent) equipped with a jet-stream ion-focus source for the UHPLC-MS analysis The UHPLC system consisted of a degasser, two binary pumps, and an autosampler. Samples of 20 mg for each of the black and white spine samples were extracted by water. Aliquots of 3 µl of filtered extract were applied to a reversed-phase column (SB-C18 RRHD, 2.1 × 100 mm, 1.8 µm; Agilent) with an in-line filter (1290 infinity in-line filter; Agilent). The system was operated in positive ion mode at a flow rate of 0.4 ml min^−1^ using solvent A (water with 0.1% formic acid) and solvent B (acetonitrile with 0.1% formic acid). The gradient started from 15% B for 3 min, followed by 15% to 19% B in 6 min, 19 % to 25% B in 2 min, 25% to 40% B in 4 min, from 40% to 100% B in 15 min, and held for 4 min, then returned to the starting condition in 1 min, keeping the re-equilibration at 15% B for 5 min. Data were collected in positive ESI mode in separate runs on a Q-TOF (Agilent 6540) operated in full-scan mode from 50 to 1000 *m*/*z*. During the analysis two reference masses (121.0509 *m/z* for C_5_H_4_N_4_, and 922.0098 *m/z* for C_18_H_18_O_6_N_3_P_3_F_24_) were continuously measured to allow constant mass correction, and to obtain accurate values. The capillary voltage was 4000 V with a scan rate of 4 s^–1^; the nebulizer gas flow rate was 12 l min^−1^; the drying gas flow was 10 l min^−1^; the gas temperature was 350 ºC, and the skimmer voltage was 65 V.

### Construction of RNA libraries for comparative transcriptomic analysis

Transcriptomic profiling was conducted using cucumber fruit spines at 2 DAA from the RNS8 and RNS9 lines and from the F_2_ population of RNS8×RNS9. Three biological replicate samples, each containing 0.2g spines from different plants, were collected. All of the spines were stored in liquid nitrogen until RNA isolation. Total RNA samples were extracted using a TIANGEN kit (DP441) according to manufacturer’s manual (Tiangen, China). The single nucleotide polymorphisms (SNPs) of the RNA-seq data for the white and black spine pools of the F_2_ population of RNS8×RNS9 were used for association analysis to map the *B* locus. mRNA was enriched using Dynabeads oligo(dT) (Dynal; NEB). After the addition of adenine, the resulting cDNAs were linked to adapters (P5, P7; NEB) and purified by gel electrophoresis. Finally, PCR products were purified (AMPure XP system) and the quality of mRNA including purity, quantity and integrity was tested using a Nanodrop, Qubit, and Agilent Bioanalyzer 2100 system based on optical density values (OD_230_, OD_260_, OD_280_), fluorescence dye binding specifically to RNA, and visual imaging. All libraries were sequenced on an Illumina HiSeq platform at Novogene (Beijing, China).

### Bioinformatics analysis of RNA-Seq data

Adaptor sequences and low-quality sequences were removed from the raw reads. Clean reads were aligned to reference genome sequences of the Cucurbit Genomics Database (http://cucurbitgenomics.org/organism/6) using Hisat2 v2.0.5.

FeatureCounts v1.5.0-p3 was used to count the reads numbers mapped to each gene, and then the FPKM of each gene was calculated based on the length of the gene and the read counts mapped to the gene.

Gene expression differences in the different sample pairs were detected using the DESeq2 R package (1.16.1). DESeq2 provides statistical routines for determining differential expression in digital gene expression data using a model based on the negative binomial distribution. The resulting *P*-values were adjusted using the Benjamini and Hochberg approach for controlling the false discovery rate (FDR). The thresholds were set using an FDR≤0.05 to determine significant differences in gene expression.

GATK2 (v3.7) software was used to perform SNP calling. Raw vcf files were filtered using the GATK standard filter method and other parameters (cluster:3; WindowSize:35; QD<2.0 o; FS>30.0; DP<10), and the SnpEff software was used for variant annotation.

### KEGG enrichment analysis of differentially expressed genes

The Kyoto Encyclopedia of Genes and Genomes (KEGG; http://www.genome.jp/kegg/) was used to identify differentially expressed genes (DEGs) in the spines of RNS8 and RSN9 lines. We used the clusterProfiler R package to test the statistical enrichment of DEGs in the KEGG pathways.

### qPCR analysis

Total RNA was prepared from spines at different developmental stages using a RNAprep pure Plant Kit (TIANGEN, Beijing, China), according to the manufacturer’s instructions. The extracted RNA was treated with RNase-free DNase I (Fermentas, Harrington, Canada) to eliminate genomic DNA contamination, according to the manufacturer’s instructions. cDNA synthesis was carried out using a RevertAid First Strand Synthesis Kit (ThermoFisher Scientific). An UltraSYBR Green Mixture qPCR kit (CWBIO) was used in the qPCR reaction to determine the expression of relevant genes using an iCycler iQ™ multicolour real-time PCR detection system (Bio-Rad). Expression of genes was normalised to that of the cucumber *actin* gene. The primers used are listed in Supplementary Table S3 at *JXB* online.

### DMACA staining

The spines were stained with 1% p-dimethylaminocinnamaldehyde (DMACA) solution (1% w/v DMACA in 1:1 ethanol:concentrated HCl) for 1 h. The stained spines were then washed in 70% (v v^−1^) ethanol for 1 h and observed under a Leica M165C microscope (Germany).

### Transient transformation in cucumber cotyledons and tobacco leaves

*CsMYB60* driven by *35S* promoter was recombined into the pCAMBIA1300 vector. The promoter of *Cs4CL* was fused with *GUS* (β-glucuronidase), and then recombined into pCAMBIA1300. The primers used are listed in Supplementary Table S3. The construct was separately transformed into *Agrobacterium tumefaciens* LBA4404. After cultivation, cells were harvested by centrifugation and resuspended in 10 mM MES buffer containing 10 mM MgCl_2_ and 200 μM acetosyringone. The OD_600_ of the *Agrobacterium* suspension was optimized at 0.6–0.8. The *Agrobacterium* suspension was then infiltrated into cotyledons of 8-d-old cucumber seedlings (Shang *et al.*, 2014) or leaves of 5-week-old tobacco (*Nicotiana benthamiana*) seedlings using a needleless syringe. After 2–3 d, the samples were harvested for GUS staining or RNA extraction.

### GUS histochemical assays

Samples were immersed into GUS staining solution [1 mM X-Gluc (5-bromo-4-chloro-3-indolyl β-d-glucuronide), 100 mM phosphate buffer pH 8.5, 0.1% v/v Triton X-100, 0.5 mM K_3_Fe(CN)_6_, 0.5 mM K_4_Fe(CN)_6_, 10 mM EDTA). After a vacuum treatment of 3 min to facilitate the penetration of the staining solution, samples were kept for 16 h in the dark at 37 °C before being destained with 70% (v/v) ethanol (Jefferson *et al.*, 1987). GUS staining was observed under a Leica M165C microscope (Germany).

### Particle bombardment

The constructed plasmids (*pro35S::CsMYB60*, *pro35S::Cs4CL*) were extracted using a PurePlasmid Midi Kit (CWBIO, China). Samples of 1 µg of the prepared plasmids were mixed with 10 µl of sterile water, 12.5 µl of 2.5 M CaCl_2_, and 5 µl of 0.1 M spermidine, and then coated onto gold particles (0.12 mg). Fruit of RNS8 at anthesis with intact white spines were subjected to particle bombardment using a biolistic PDS1000/He Particle Gene Gun System (Bio-Rad). Each target was bombarded at least three times. The bombarded fruits were placed on moisturized filter paper in glass dishes for 6 d.

### McrBC-based methylation assays

Genomic DNA samples (500 ng each) were digested for 2 h at 37 °C with 30 U of McrBC enzyme (NEB). A mock digestion was performed in parallel with no enzyme. Then, 5 ng DNA from each sample was used for PCR analysis. DNA was extracted using a Plant Genomic DNA Kit (TIANGEN, China).

### Bisulphite sequencing

Samples of ~2 μg of genomic DNA were used for bisulphate conversion. Bisulphite modification and desulfonation of genomic DNA were performed using a DNA Bisulfite Conversion kit (TIANGEN) according to the manufacturer’s instructions. The bisulphite-treated DNA was amplified using Taq DNA polymerase. The thermal cycling program was 95 °C for 4 min followed by 35 cycles of 95 °C for 30 s, annealing for 30 s, and extension at 72 °C for 45 s, ending with a 10 min extension at 72 °C. PCR products were cloned into the pMD18-T vector (TaKaRa), and 10 individual clones were sequenced. DNA cytosine methylation in the CG, CHG, and CHH contexts was analysed and displayed using the CyMATE software (http://www.cymate.org/).

### Generation of alignment files

CyMATE does not align sequences itself but reads pre-aligned sequence data. As all sequences must be of equal length (‘blunt-ended’ alignment), leading and trailing gaps should be inserted if necessary. Alignments containing the range between the primers in the master sequence followed by the sequences of individual clones in the desired order were generated using CLUSTAL version 1.83 (http://www.ebi.ac.uk/clustalw). The alignments were saved in sequential (standard FASTA) and interleaved formats (standard CLUSTAL).

## Results

### Phenotypic characteristics of white and black spines

**Fig. 1.**
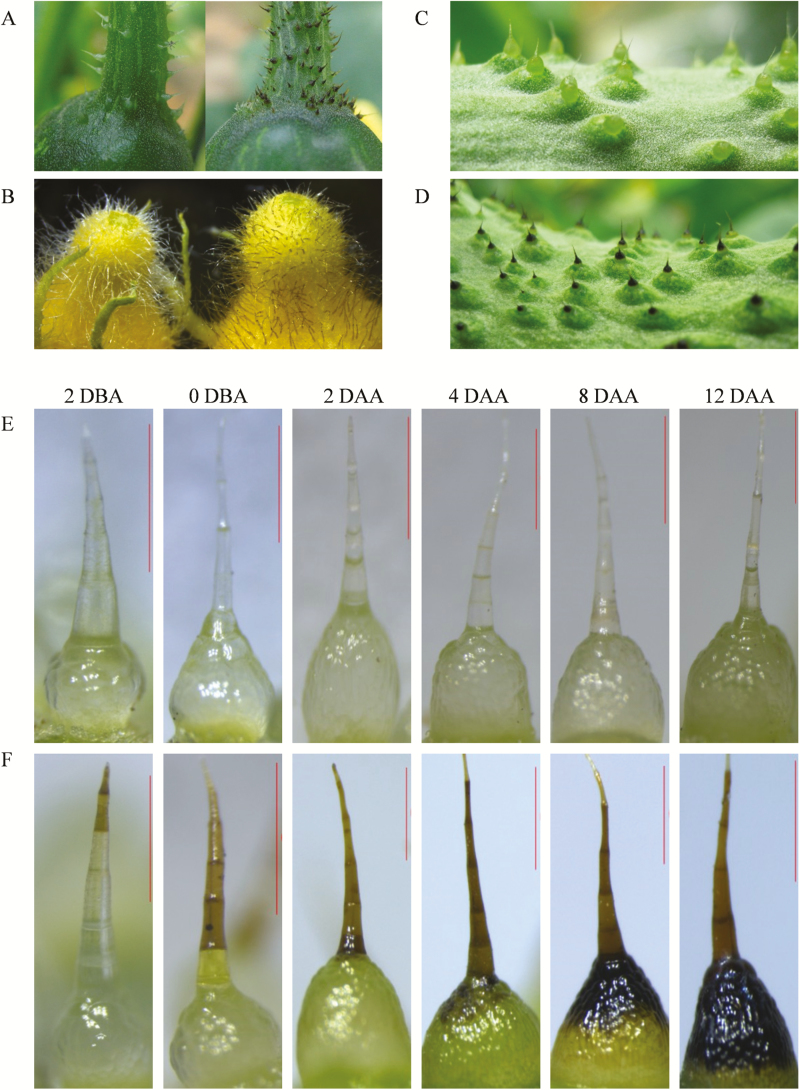
Phenotypic comparison of organs with accumulated pigments between white-spine (RNS8) and black-spine (RNS9) cucumber inbred lines. (A) Pedicel, (B) Receptacle, (C) white spines on the fruit skin, and (D) black spines on the fruit skin. (E) No pigmentation of white spines from 2 d before anthesis (DBA) to 12 d after anthesis (DAA) in RNS8. (F) Pigmentation time course of black spines from 2 DBA to 12 DAA in RNS9. Scale bars are 0.7 mm.

The colour of the fruit spine is an important quality trait for cucumber. To explore the pigments in black spines, we used two inbred lines with a similar plant stature and fruit shape, RNS8 with white spines and RNS9 with black (the colours can be distinguished easily on the developing young fruit after anthesis). The pigments in black spines exhibited a tissue-specific pattern and could also be observed on the fruit surface and in trichomes of receptacles and pedicels (Fig. 1A–D, Supplementary Fig. S1).

The black colour appeared on the very top cells of the spines at approximately 2 d before anthesis (DBA). The colouration then gradually moved downward from 4–12 days after anthesis (DAA). The whole spine became completely black at 12 DAA (Fig. 1F). In contrast, white spines showed no significant changes in colour (Fig. 1E).

Previous studies have indicated that an orange colour of mature fruit is associated with black spine colour, and both of these traits are controlled by a single dominant gene, *B* (Li *et al.*, 2013). This association was also observed in the present study (Supplementary Fig. S1).

### Gross comparisons of metabolite profiles between white and black spines

**Fig. 2.**
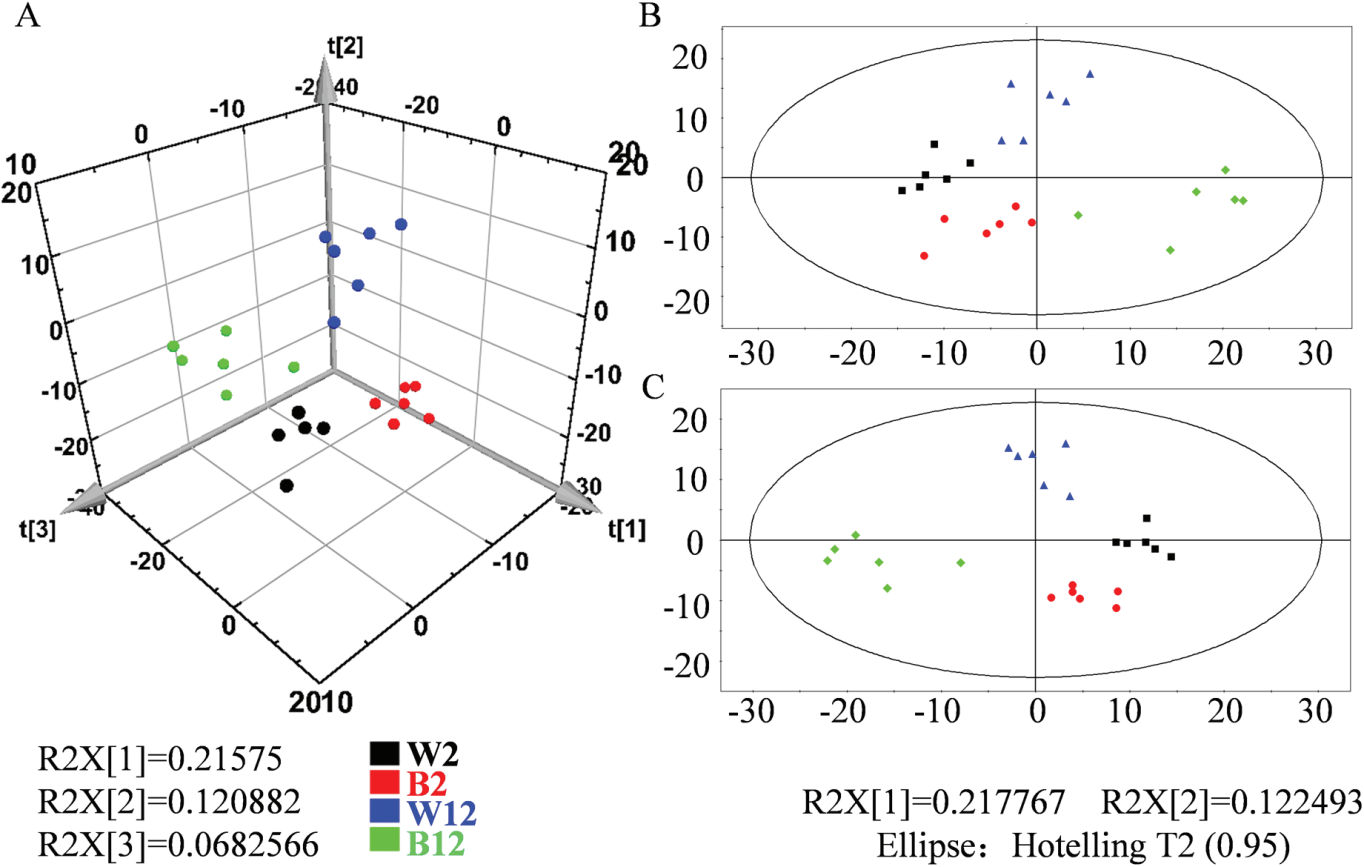
Principal component analysis (PCA) and Partial Least Squares Discrimination Analysis (PLS-DA) of metabolite profiling data for comparison of white spines (line RNS8) and black spines (line RNS9) in cucumber. (A) PLS-DA three-dimensional stereogram, (B) PCA plane figure, (C) PLS-DA plane figure. W2, white spine at 2 DAA; B2, black spine at 2 DAA; W12, white spine at 12 DAA; B12, black spine at 12 DAA.

Abundant metabolites that distinguished between white and black spines could be recognized by visual inspection of LC-MS chromatograms and through the use of statistical methods such as principal component analysis. As shown in Fig. 2, slight differences in metabolites began to emerge between the white (RNS8 line) and black (RNS9 line) spines at 2 DAA, and these had become significant by 12 DAA. The results thus indicated that the metabolites were mainly synthesized in the late developmental stage. A total of 115 different metabolites were detected between the white and black spines at 2 DAA, and 135 different metabolites at 12 DAA (Supplementary Fig. S2, Supplementary Table S1).

### Isolation and identification of flavonols in black spines

To determine the secondary metabolites in black spines, we performed chromogenic reactions on water extracts using different chemical agents (Supplementary Fig. S3). The results indicated that no anthocyanins were present in black spines because none of the relevant colour alterations occurred when the pH value was changed through the addition of acid or alkali to water extracts from black spines. Consistently, no anthocyanins were detected in the metabolic analysis (Supplementary Table S1). However, the chromogenic reaction with FeCl_3_ showed an accumulation of phenolic compounds in the black spines (Supplementary Fig. S3).

**Fig. 3.**
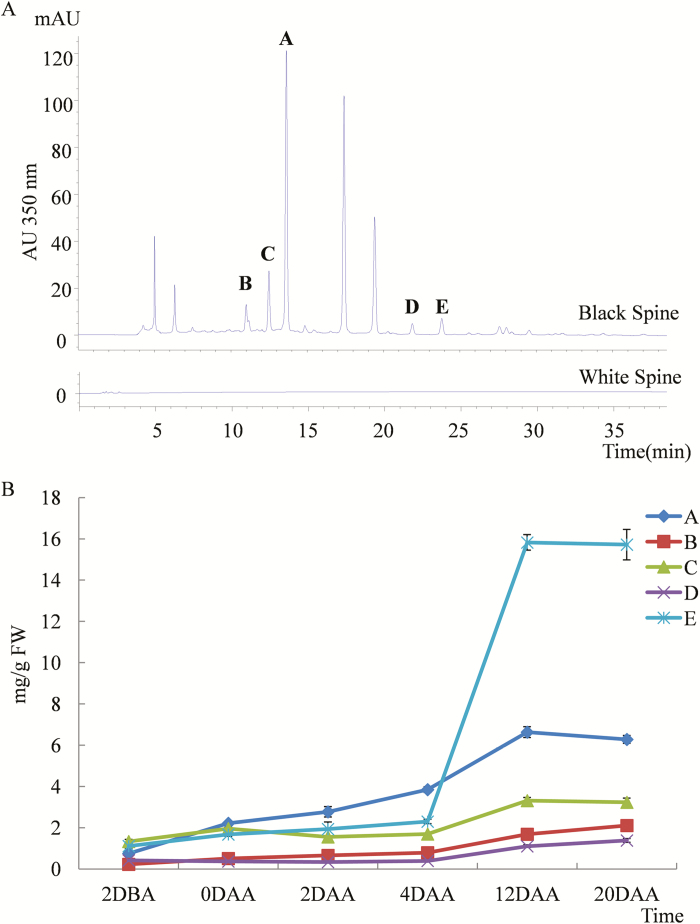
Composition and content of flavonols in black spines of the cucumber line RNS9 compared with the white spines of line RNS8. (A) Flavonol HPLC signals were recorded in black spines but not in white spines. The peaks A–E indicate five different metabolites that could be purified for subsequent NMR. (B) The contents of metabolites A–E in black spines at different developmental stages. Data are means (±SD) (*n*=3). DBA, days before anthesis; DAA, days after anthesis.

**Fig. 4.**
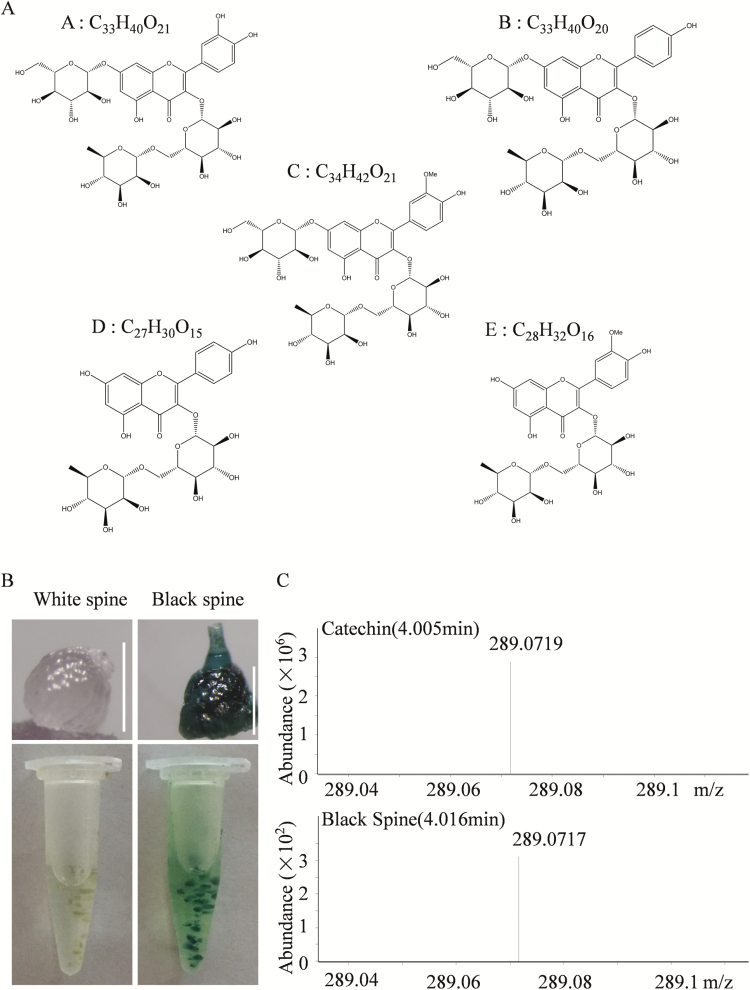
Molecular formulae and structures of the five flavonols identified by NMR, and identification of PAs in black spines of line RNS9. (A) The formulae and structures of A, quercetin-3-O-rutinoside -7-O-glucose; B, kempferol 3-O-rutinoside 7-O-glucoside; C, isorhamnetin -3-O-rutinoside-7-O-glucoside; D, kaempferol -3-O- rutinoside; and E, isorhamnetin- 3-O-rutinoside. The NMR physicochemical and spectral data are listed in Supplementary Fig. S5. (B) DMACA (p-dimethylaminocinnamaldehyde) staining of white and black spines (white spines of the RNS8 line). The images show single stained spines (top) and many stained spines (bottom). Scale bars are 1 mm. (C) Confirmation of proanthocyanidins in black spines as determined by tandem MS using catechin as a control. The MS ion peak signal for the catechin monomer is shown (*m*/*z* 289.0718 ± 5 ppm).

Using HPLC for flavonols, several distinct metabolites were separated from black spines that were not present in white spines (Fig. 3A). Five of these (designated as A–E) were present in sufficient quantity to be purified for parsing of their molecular structures using NMR analysis. The results indicated that the metabolites were quercetin-3-O-rutinoside-7-O-glucose (A), kempferol-3-O-rutinoside-7-O-glucoside (B), isorhamnetin-3-O-rutinoside-7-O-glucoside (C), kaempferol-3-O-rutinnoside (D), and isorhamnetin-3-O-rutinoside (E) (Fig. 4A). The physicochemical and spectral data are given in Supplementary Fig. S5. The NMR results were also verified by data obtained by high-resolution MS (Supplementary Fig. S6). In agreement with the progression of colouration, these five flavonols accumulated gradually as the spines developed from 2 DBA to 20 DAA in black spines (Fig. 3B, Supplementary Fig. S4). At 12 DAA, isorhamnetin-3-O-rutinoside was present at the highest concentration, followed by quercetin-3-O-rutinoside-7-O-glucose, isorhamnetin-3-O-rutinoside-7-O-glucoside, kempferol-3-O-rutinoside rutinoside-7-O-glucoside, and kaempferol-3-O-rutinnoside.

### Identification of proanthocyanidins in black spines

Proanthocyanidins (PAs; also called condensed tannins) are oligomers of flavan-3-ol units (catechin and epicatechin) and are prominent compounds in seed coats, leaves, fruit, flowers, and bark of many plant species (Dixon *et al.*, 2005). DMACA staining is a rapid and effective method for histological analysis of PAs (Xie and Dixon, 2003; Peng *et al.*, 2012), and it indicated that black spines had high accumulation of PAs, whereas white ones did not (Fig. 4B). The presence of PAs in black spines was further confirmed by MS ion peak signals for catechin monomers (*m*/*z*, 289.0718 ± 5 ppm) (Fig. 4C).

### Transcriptomic profiles of fruit spines

To investigate the molecular basis underlying the difference in colour between white and black spines, a comparative transcriptomic analysis was performed using spines from the lines RNS8 (white) and RNS9 (black) at 2 DAA. High-throughput RNA sequencing (RNA-Seq) produced 52, 56, and 54, million reads for the three white-spine samples and 57, 71, and 48 million reads for the black-spine samples (Supplementary Table S2). In addition, SNPs with high confidence were identified using GATK2 (v3.7). Through SNP association analysis, the *B* gene was located on the distal region of the short arm of chromosome 4, which contains a R2R3-MYB gene, *CsMYB60* (Zhao *et al.*, 2014) (Supplementary Fig. S7). This result is consistent with that of a previous study (Li *et al.*, 2013).

**Fig. 5.**
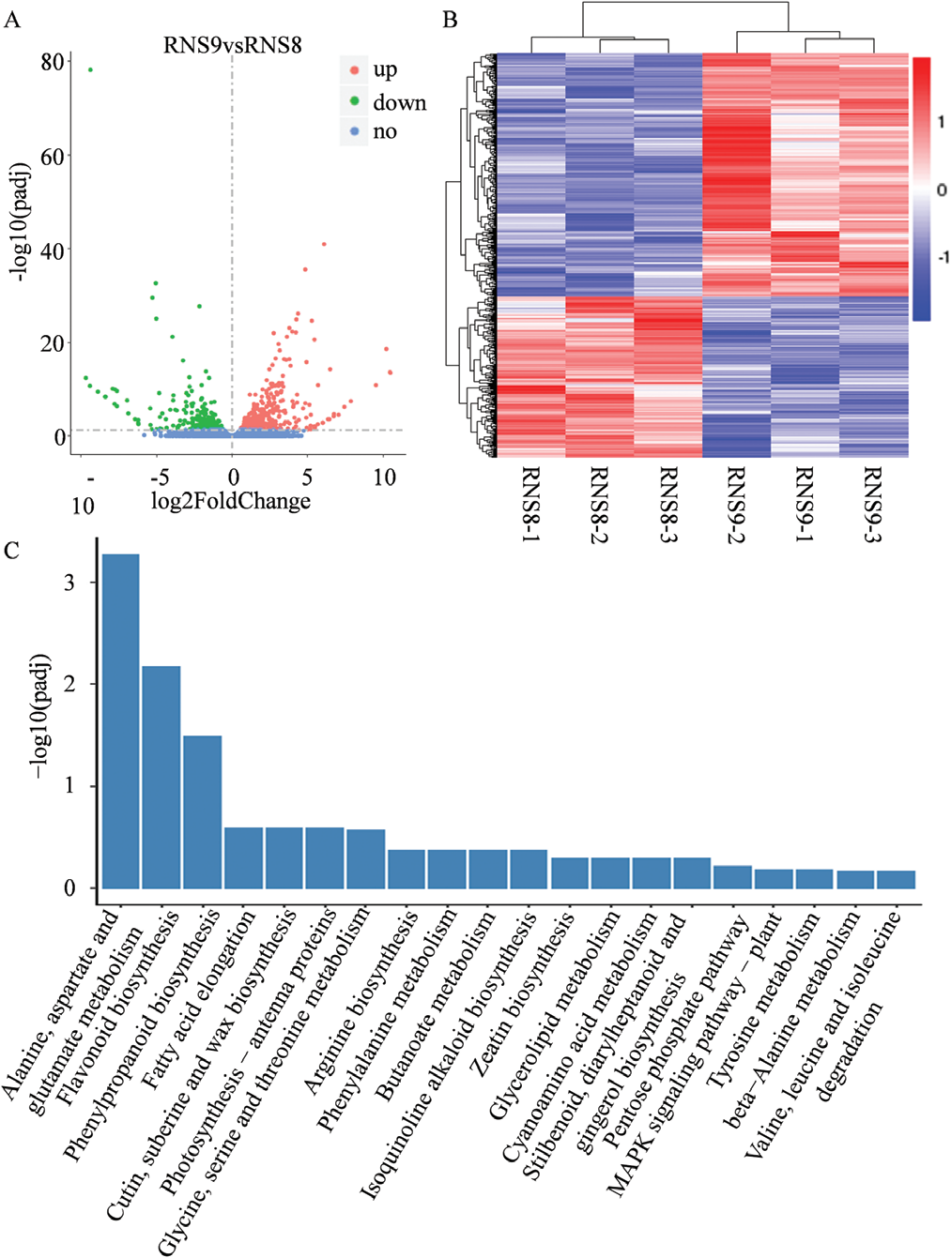
Transcriptomic analysis of cucumber fruit spines. (A) Volcano plot showing the differentially expressed genes (DEGs) between RNS8 (white spines) and RNS9 (black spines) (*P*<0.05). (B) Cluster analysis of DEGs. Genes with high or weak expression in the RNS8 vs. RNS9 group fruit spines are shown in red and blue, respectively. (C) Significantly enriched KEGG pathways (P <0.05) in the black fruit spines compared with white spines at 2 d after anthesis.

We found 743 differentially expressed genes (DEGs) (Supplementary Table S4) between white and black spines, of which 447 were up-regulated and 296 were down-regulated in the white spines compared with the black (Fig. 5A, B). KEGG analysis using a hyper-geometric distribution test revealed pathways that were closely associated with the metabolic process and showed significant differences between the white and black spines. The significantly enriched pathways included ‘Flavonoid biosynthesis’, ‘Phenylpropanoid biosynthesis’ and ‘Phenylalanine metabolism’ (Fig. 5C), which was in accordance with our other results.

### Polymorphisms in the DNA sequence of *CsMYB60* in different cucumber varieties

Li *et al* (2013) mapped the *B* gene to a 50-kb region containing a R2R3-MYB gene on cucumber chr.4. This result was verified by SNP association in this study (Supplementary Fig. S7). The R2R3-MYB gene, *CsMYB60* (Zhao *et al.*, 2014), is considered as the best candidate for the *B* locus (Li *et al.*, 2013). To understand the evolutionary relationship between CsMYB60 and Arabidopsis R2R3-MYB proteins, phylogenetic analysis was also performed using the neighbor-joining method (Supplementary Fig. S8). This showed that CsMYB60 is closely related to AtMYB111, AtMYB11, and AtMYB12, which regulate the early biosynthetic genes of flavonoids in Arabidopsis, suggesting that *CsMYB60* may be involved in flavonoid biosynthesis in cucumber. We compared the sequences of the two *CsMYB60* alleles from the inbred lines RNS8 (white spines) and RNS9 (black spines), and found a 6994-bp insertion at the 1492-bp position after the transcription start site in the second intron of *CsMYB60* in RNS8 compared with RNS9. To determine whether this R2R3-MYB gene was conserved in different varieties, we tested 20 other cucumber inbred lines from different geographical regions that were available in our laboratory (Zhang *et al.*, 2016); 14 of the 22 lines have white spines and eight have black spines. The results of the sequencing analysis indicated that the *CsMYB60* alleles from the lines with black spines (CNS14, CNS20, RNS5, RNS12, and RNS13) had the same genomic sequence as that in RNS9. In addition, the sequence of *CsMYB60* in wild cucumber in the Cucurbit Genomics Database (http://cucurbitgenomics.org/organism/6) is similar to that of RNS9, with the exception of several SNPs that do not affect the ORF. The 14 inbred lines with white fruit spines could be classified into two groups based on the variation type: one group was formed of CNS13, CNS16, CNS17, CNS18, and CNS23 and had a SNP (C to T), which leads to a premature stop codon yielding a truncated protein product, in the second exon of *CsMYB60* in comparison to RNS9; the other group, was formed of CNS5, CNS10, CNS15, CNS22, RNS2, RNS3, RNS8, RNS10, and ‘Chinese long’ 9930, and had a 6994-bp insertion at the 1492-bp position after the transcription start site in the second intron of *CsMYB60*. Interestingly, the lines RNS14 and RNS15 had two inserts (a 2759-bp fragment at 853 bp and a 6994-bp fragment at 1492 bp after the transcription start site) in the second intron of *CsMYB60*, but still displayed the black spine phenotype (Table 1).

**Table 1.** Polymorphism of the DNA sequence of *CsMYB60* in different cucumber lines relative to black-spined RNS9.

Lines	Colour of spine	Polymorphism of DNA
CNS5	White	6994-bp insertion in second intron at 1492bp after TSS
CNS10	White	6994-bp insertion in second intron at 1492bp after TSS
CNS15	White	6994-bp insertion in second intron at 1492bp after TSS
CNS22	White	6994-bp insertion in second intron at 1492bp after TSS
Chinese long 9930	White	6994-bp insertion in second intron at 1492bp after TSS
RNS2	White	6994-bp insertion in second intron at 1492bp after TSS
RNS3	White	6994-bp insertion in second intron at 1492bp after TSS
RNS8	White	6994-bp insertion in second intron at 1492bp after TSS
RNS10	White	6994-bp insertion in second intron at 1492bp after TSS
CNS13	White	SNP (C to T) at 617 bp after TSS, Arg to stop codon
CNS16	White	SNP (C to T) at 617 bp after TSS, Arg to stop codon
CNS17	White	SNP (C to T) at 617 bp after TSS, Arg to stop codon
CNS18	White	SNP (C to T) at 617 bp after TSS, Arg to stop codon
CNS23	White	SNP (C to T) at 617 bp after TSS, Arg to stop codon
CNS14	Black	No change
CNS20	Black	No change
RNS5	Black	No change
RNS12	Black	No change
RNS13	Black	No change
RNS14	Black	2759-bp insertion in second intron at 853bp after TSS 6994-bp insertion in second intron at 1492bp after TSS
RNS15	Black	2759-bp insertion in second intron at 853bp after TSS 6994-bp insertion in second intron at 1492bp after TSS

TSS, transcription start site.

### Decreased expression level of *CsMYB60* and the key genes involved in flavonoid biosynthesis in white fruit spines

**Fig. 6.**
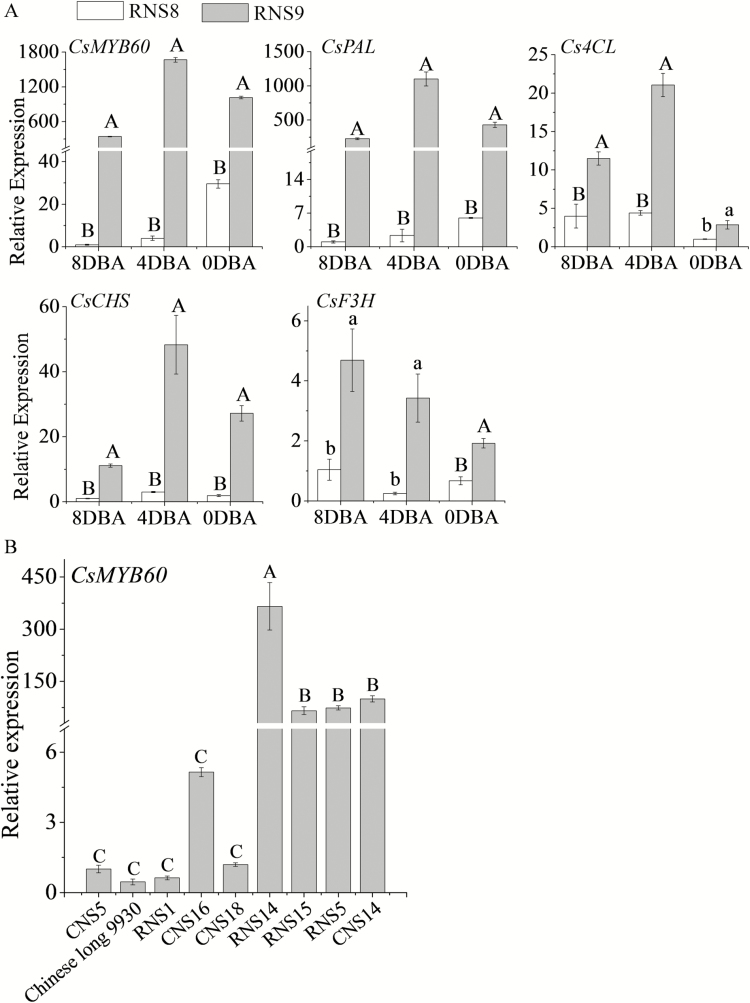
Expression of *CsMYB60* and key genes involved in flavonoid biosynthesis in cucumber fruit spines of the lines RNS8 (white spines) and RNS9 (black spines). All data are means (±SD) of three technical replicates. (A) Expression of genes in spines at 8 d before anthesis (DBA), 4 DBA, and 0 DBA. Expression values are relative to that of the cucumber *actin* gene. Different capital letters indicate significant differences at *P*<0.01and different lowercase letters indicate differences at *P*<0.05. The other two biological repeats are shown in Supplementary Fig. S9A. (B) The expression of CsMYB60 in spines at 0 DAA from different inbred lines. The other two biological repeats are shown in Supplementary Fig. S8B. Different letters indicate significant differences at P<0.01 Significant differences were determined according to a multiple statistical analysis system using Data Processing System (DPS) statistics version 7.05 software.

The 6994-bp insertion at the 1492-bp position did not knock out *CsMYB60* but dramatically decreased its expression level at different developmental stages of spines in white-spined RNS8. Moreover, the expression levels of the key structural genes in the synthetic pathway of flavonoids, such as *CsPAL*, *Cs4CL*, *CsCHS*, and *CsF3H*, also decreased in at least one of the three spine developmental stages tested (8, 4, and 0 DBA) in RNS8 compared with black-spined RNS9 (Fig. 6A, Supplementary Fig. S9A).

To further test the importance of *CsMYB60* in spine pigmentation, we selected five additional white spine lines (Chinese long 9930, RNS1, CNS5, CNS16, CNS18) and four black spine lines (RNS5, RNS14, RNS15, CNS14) with different *CsMYB60* alleles (Table 1) for expression level analysis. The results indicated that the gene expression level of *CsMYB60* was much higher in the black spine lines than in the white spine lines (Fig. 6B, Supplementary Fig. S9B).

### Overexpression of *CsMYB60* and *Cs4CL* induces pigmentation of white spines

**Fig. 7.**
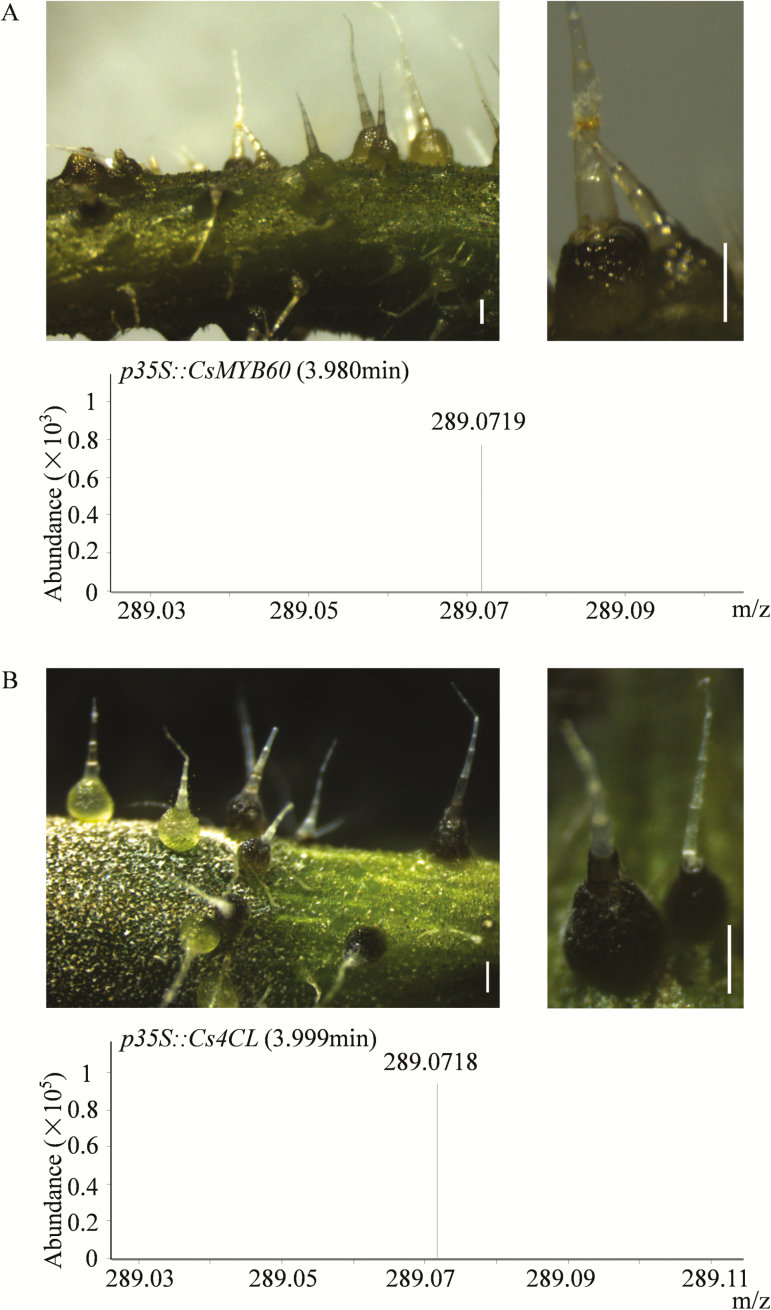
Overexpression of *CsMYB60* and *Cs4CL* induces pigmentation of white spines in cucumber. (A) *CsMYB60* transformation mediated by particle bombardment in white spines of the line RNS8, with confirmation of proanthocyanidins (PAs) in the transgenic spines as determined by tandem MS. (B) *Cs4CL* transformation mediated by particle bombardment in white spines of RNS8, with confirmation of PAs in the transgenic spines as determined by tandem MS. The MS ion peak signals for the catechin monomer are *m*/*z* 289.0718 ± 5 ppm. All scale bars are 0.7 mm.

If *CsMYB60* is the *B* locus, then increasing its expression level in white spines should lead to pigment accumulation. We tested this hypothesis using a transient expression system, and found that overexpression of *CsMYB60* could indeed transform white spines into black ones. The presence of PAs in the spines overexpressing *CsMYB60* was further confirmed by MS analysis (Fig. 7A). In addition, using this transient expression system, we also assessed the limiting structural gene in the synthetic pathway of flavonoids. We examined the *CsPAL*, *CsCHS*, *Cs4CL*, *CsF3H*, and *CsGST* genes involved in the flavonoid pathway. Interestingly, at least under our experimental conditions, only *Cs4CL* could change white spines into black ones, in which the presence of PAs was also confirmed by tandem MS (Fig. 7B).

### *CsMYB60* can indirectly activate *Cs4CL* expression

**Fig. 8.**
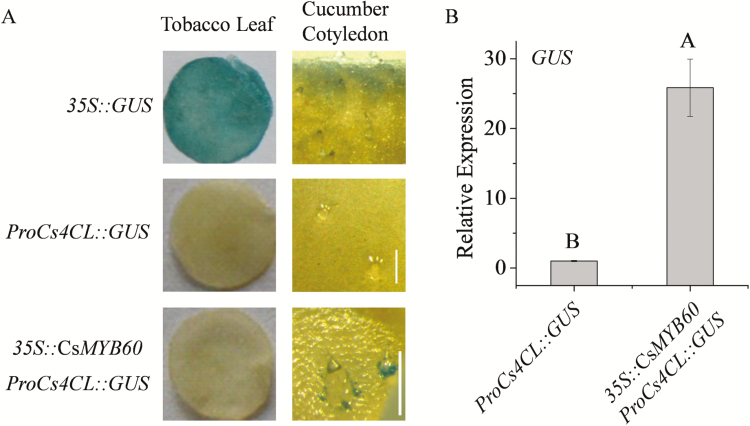
Expression of *Cs4CL* can be activated indirectly by overexpressed *CsMYB60* in cucumber cotyledons. (A) GUS staining assays in *Nicotiana benthamiana* leaves and cucumber cotyledons for *proCs4CL::GUS* transfection, and *pro35S::CsMYB60* and *proCs4CL::GUS* co-transfection. Scale bars are 0.25 mm. (B) Relative expression of GUS in cucumber cotyledons transfected with *proCs4CL::GUS*, and *pro35S::CsMYB60* plus *proCs4CL::GUS*. Data are means (±SD) of three technical replicates. The other two biological repeats are shown in Supplementary Fig. S10. Different letters indicate significant differences at *P*<0.01. Significant differences were determined according to a multiple statistical analysis system using Data Processing System (DPS) statistics version 7.05 software.

In our study, overexpression of *CsMYB60* or *Cs4CL* could lead to the synthesis of PAs and flavonols in white spine plants (Fig. 7). Therefore, we used *Agrobacterium*-mediated transient transformation of cucumber cotyledons and tobacco leaves to clarify their relationship. GUS staining showed that ectopic expression of *CsMYB60* could activate the *Cs4CL* promoter in cucumber cotyledons, but not in tobacco leaves (Fig. 8A). The gene expression level of *GUS* was significantly higher in the cucumber cotyledons that were co-transfected with *35S::CsMYB60* and *proCs4CL::GUS* than in those transfected with only *proCs4CL::GUS* (Fig. 8B, Supplementary Fig. S10). The results indicated that *CsMYB60* could indirectly activate *Cs4CL* expression in cucumber. Thus, as a key regulatory gene, *CsMYB60* might cooperate with other factors to indirectly activate the expression of *Cs4CL,* leading to the synthesis of PAs and flavonols in black spines.

### Identification of a cucumber *Mutator*-like element

**Fig. 9.**
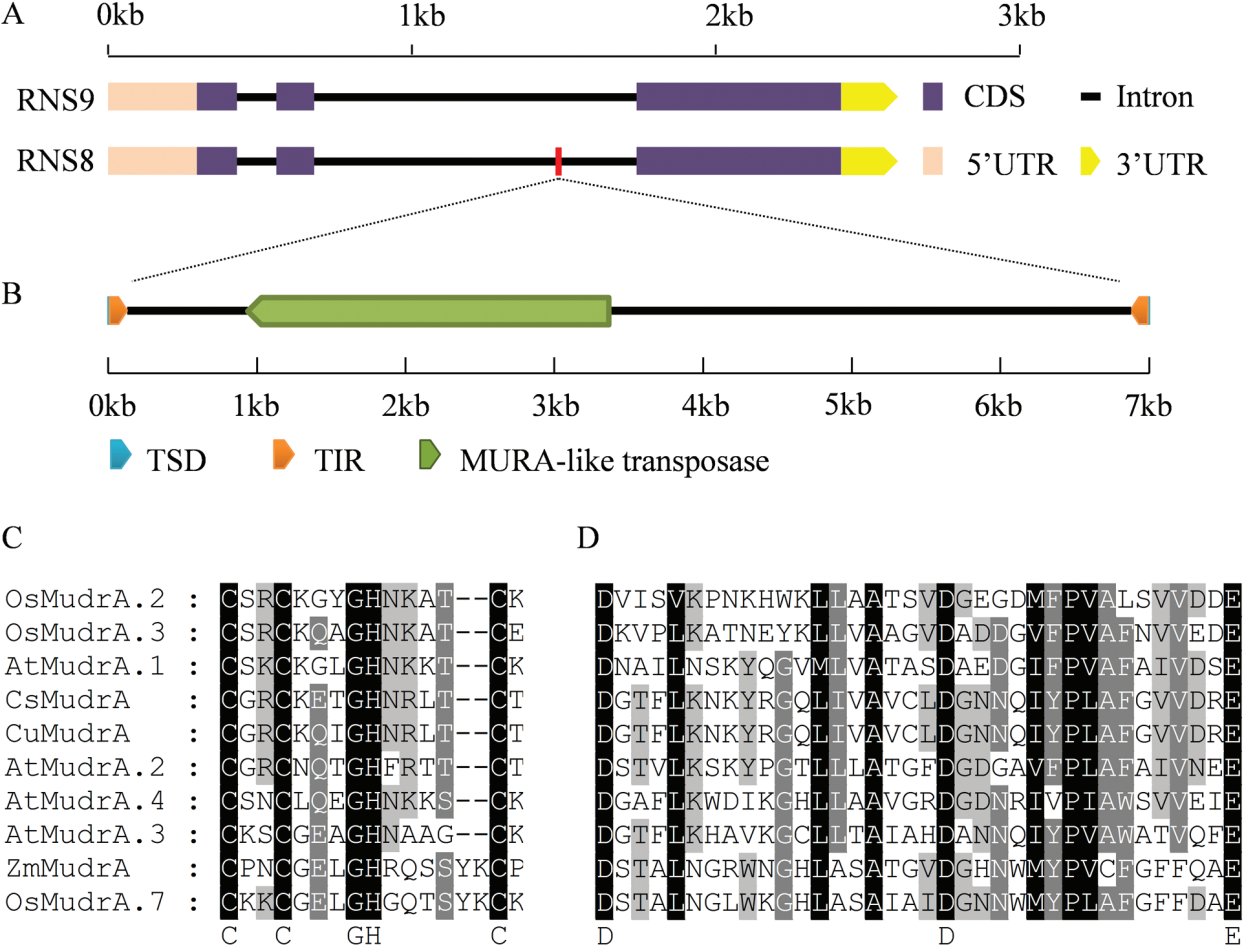
The inserted fragment in *CsMYB60* is a *Mutator*-like element (MULE) in the white-spined cucumber line RNS8. (A) Gene structure of *CsMYB60* alleles from RNS9 (black spines) and RNS8 (white spines). (B) Schematic diagram of CsMULE with *MURA*-like transposase, terminal inverted repeats (TIRs), and target site duplications (TSDs). (C) Sequence alignment of the CX2CX4HX4(or 6)C motif of the *mudrA*-like protein in CsMULE. (D) Sequence alignment of the DX18DX15E motif of the *mudrA*-like protein in CsMULE. Zm, *Zea mays*; Cs, *Cucumis sativus*; Cu, *Cucumis melo*; At, *Arabidopsis thaliana*; Os, *Oryza sativa*. The GenBank accession numbers of the proteins used are listed in Supplementary Fig. S11.

Transposable elements (TEs) play vital roles in generating genomic novelty and diversity in plants (Song and Cao, 2017). We were interested in whether the 6994-bp insertion in the second intron of *CsMYB60* in RNS8 was a TE (Fig. 9A) and, if so, of what type. We therefore analysed the fragment and found that it contained a pair of terminal inverted repeats (TIRs; left 117-bp TIR, right 115-bp TIR) and a pair of 9-bp target site duplications (TSDs) flanking each of the two TIRs, resulting in a total length of 7003 bp (Fig. 9B). The blastn search results showed that the insertion also included a MuRA-like transposase gene. Phylogenetic analysis of CsMudrA homologues from several species revealed that CsMudrA shares the highest similarity with CuMudrA (Supplementary Fig. S11). These results indicated that this insertion in *CsMYB60* from white-spined RNS8 is a *Mutator*-like element (MULE), which we named CsMULE (GenBank: MG558001).

A conserved motif, CX2CX4HX4(or6)C (where X represents any amino acid), characteristic of DNA binding domains, has been identified at the C-terminal end of MuRA from Arabidopsis (Yu *et al.*, 2000). We also found this conserved motif in the *MudrA*-like gene in CsMULE (Fig. 9C). The transposons of eukaryotic organisms generally have a characteristic triad of acidic amino acids, D-D-E, in the *Mutator*-like transposase region. The motif conforms to DX18DX15E, with 35 amino acids intervening between the first D and E (Rossi *et al.*, 2004). Functional analyses have shown that mutations in these conserved amino acids prevent mobilization of the element, demonstrating that these residues are part of the transposase active site (Rossi *et al.*, 2004). We again found the characteristic motif of DX18DX15E in the *MudrA*-like gene in CsMULE (Fig. 9D). These results further confirmed that the insertion in *CsMYB60* was the MULE in cucumber.

### Insertion of CsMULE decreases *CsMYB60* expression levels in an epigenetic manner

**Fig. 10.**
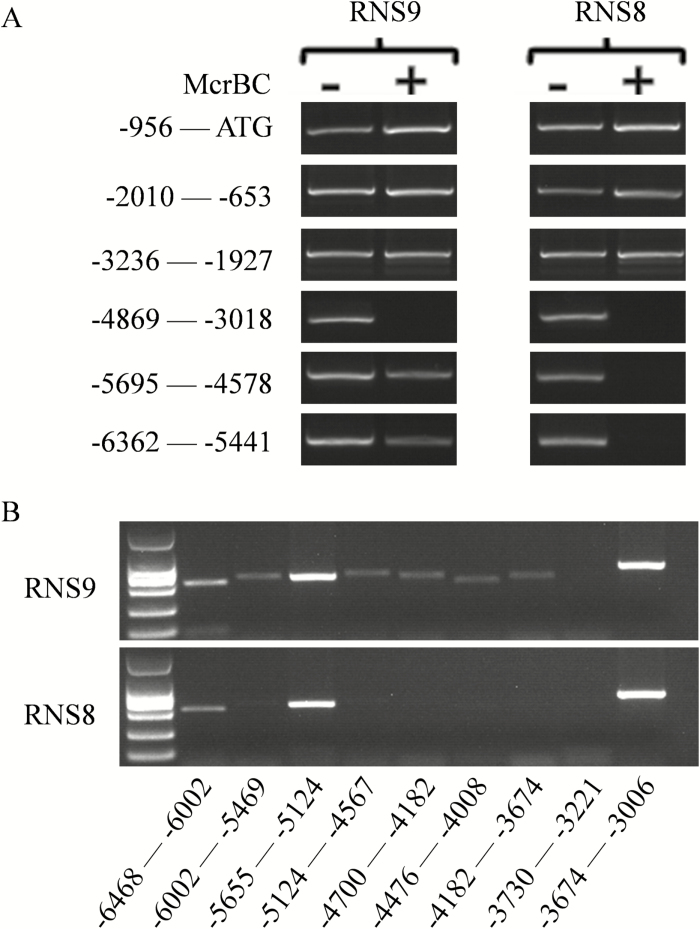
DNA methylation status of the *CsMYB60* promoter in cucumber fruit with white and black spines. (A) McrBC-sensitive PCR analysis of the promoter of *CsMYB60* in RNS8 (white spines) and RNS9 (black spines). + and – indicate whether or not the genomic DNA was treated with McrBC before PCR amplification. (B) McrBC-sensitive PCR analysis of the region from –6468 to about –3006 before the translational start codon (ATG) of *CsMYB60* in RNS8 and RNS9. The absence of a PCR product in McrBC-treated samples indicates methylated DNA.

Transposons can lead to DNA methylation that alters gene expression (Martin *et al.*, 2009). Since the insertion in *CsMYB60* in RNS8 was a *Mutator*-like transposon, we were interested to determine whether the decrease in *CsMYB60* expression was caused by this CsMULE in an epigenetic manner. Therefore, we analysed the DNA methylation status of the *CsMYB60* promoter using a McrBC-sensitive PCR analysis and observed a much higher methylation level of the –6362 to –4578 promoter region of *CsMYB60* in white-spined RNS8 than in black-spined RNS9 (Fig. 10A). Next, we divided the region into nine sub-sections and performed PCR analysis with genomic DNA treated with McrBC as a template (Fig. 10B). We then selected two regions of the *CsMYB60* promoter for methylation analysis using bisulphite sequencing (Supplementary Fig. S12). The results indicated that several sub-sections had higher methylation in RNS8 than in RNS9. Thus, the insertion of CsMULE might cause greater methylation of the *CsMYB60* promoter, thus decreasing its expression level in white-spined RNS8.

## Discussion

The visual appearance of cucumber fruit is a highly important trait for varietal improvement (Chen *et al.*, 2016). The spine colour is a fruit-related characteristic and is considered an important factor for the economic value of the fruit (Li *et al.*, 2013). Cultivated cucumber was domesticated from its wild form *Cucumis sativus var. hardwickii*, which still grows in the Himalayan foothills in India, Myanmar, north and west Thailand, and southwest China (Qi *et al.*, 2013; Renner, 2017). Most cultivated cucumbers, especially slicing cucumbers, have white spines, but the wild cucumber has black spines (Qi *et al.*, 2013; Renner, 2017). In this study, we determined that the pigments of black spines mainly comprise flavonols and PAs (Fig. 4A, C). A previous study showed that epidermal flavonoids, which absorb UV-radiation, protect the internal tissues of leaves and stems (Treutter, 2006). In addition, an inheritance study using groundnut provided good evidence for quercetin and rutin having a significant negative impact on the development and mortality of neonate larvae of *Spodoptera litura* in interspecific derivatives of *Arachis* species (Mallikarjuna *et al.*, 2004). PAs that are present in the fruit, bark, leaves, and seeds of many plants can provide protection against predation and disease (Dixon *et al.*, 2005). Moreover, both the traits of orange skin in mature fruit and black spines are controlled by a single dominant gene (Li *et al.*, 2013) (Supplementary Fig. S1). Thus, as a result of natural selection, the flavonoid pigments in black spines and fruit skin should provide protection for the seeds of wild cucumber. Flavonoids in various plants are beneficial not only for the plant itself as physiologically active compounds or as stress-protecting agents (Treutter, 2006) but also for humans as antioxidants (Qin *et al.*, 2008). Therefore, our present findings might provide the impetus for a new direction of breeding to obtain increasing flavonoids in the fruit endocarp of cucumber to benefit human health.

Genetic analysis has indicated that the black fruit-spine trait is controlled by a single gene, *B*, and is dominant over the white trait (Walters *et al.*, 2001; Li *et al.*, 2013). A R2R3-MYB gene on cucumber chr.4 has been considered the best candidate for the *B* locus (Li *et al.*, 2013), and this gene was named *CsMYB60* (Zhao *et al.*, 2014). In our present study, through SNP association, we also mapped *B* to the same region of chr.4 containing *CsMYB60* (Supplementary Fig. S7), and we found that all the tested inbred lines with white spines had an altered allele and reduced expression of *CsMYB60* (Table 1; Fig. 6B). Finally, overexpression of *CsMYB60* could transform white spines into black spines (Fig. 7A). These findings indicate that *CsMYB60* can be identified as the *B* locus.

Control of the flavonoid pathway is an ancestral function of the MYB-bHLH-WD40 (MBW) complex in plants (Xu *et al.*, 2015*a*). In Arabidopsis, the MBW complex activates late flavonoid biosynthetic genes to control the production of anthocyanins and PAs, whereas early flavonoid biosynthetic genes are activated by three R2R3-MYB proteins (MYB11, MYB12, and MYB111) (Li, 2014). Flavonoid MBW proteins might be conserved in cucumber, but the mechanisms regulated by the MBW complex and/or by MYB proteins may differ from those in Arabidopsis and other plants (Li, 2014; Xu *et al.*, 2015*a*) because our data showed that *Cs4CL*, the last structural gene in general phenylpropanoid metabolism, is the limiting gene for flavonoid biosynthesis in cucumber and is activated indirectly by *CsMYB60* (Fig. 7B; Fig. 8). To our knowledge, this kind of regulatory mechanism has not been reported before. Further studies are needed to clarify the detailed mechanisms responsible for flavonoid biosynthesis.

*Mutator* transposons are the most ubiquitous DNA transposable elements in plant genomes and play a special role in gene and genome evolution (Diao and Lisch, 2006). *Mutator* activity can result in a wide range of changes, including changes in gene expression (Raizada *et al.*, 2001). In our study, the insertion in the second intron of *CsMYB60* in white-spined RNS8 was found to be a CsMULE based on its typical *Mutator* characteristics (Fig. 9C, D). The decreased expression level of *CsMYB60* might have been caused by this CsMULE in an epigenetic manner because the methylation level of a particular region of the *CsMYB60* promoter was much higher in the white-spine inbred line than in the black (Fig. 10). This result is in agreement with a previous study of the transition from male to female flowers in gynoecious melon, where the natural and heritable epigenetic change resulted from the insertion of a transposon that is required for the initiation and maintenance of the spreading of DNA methylation to the *CmWIP1* promoter (Martin *et al.*, 2009). Transposon-mediated epigenetic regulation in gene expression also occurs in rice (Song and Cao, 2017).

The transposon *Mutator* has been recognized as being one of the most active mobile elements in plants and has been widely employed as a mutagenizing system (van Leeuwen *et al.*, 2007). For instance, *RescueMu* has been used for high-throughput gene mutation and cloning (Raizada *et al.*, 2001; John *et al.*, 2004). Through the *Mutator* transposon system, many genes have been successfully cloned, such as *An1*, *Zag1*, and *Fl1* in maize (May *et al.*, 2003; Raizada, 2003). Thus, the CsMULE identified in our present study might also be used in the future as a mutagenizing tool for functional genomics in cucumber.

In conclusion, we determined that the pigments of black fruit spines in cucumber are primarily composed of flavonols and PAs. *CsMYB60* is a key regulatory gene, and *Cs4CL* is activated indirectly by *CsMYB60* as a limiting structural gene in the flavonoid biosynthesis pathway. In addition, the CsMULE insertion might result in decreased *CsMYB60* expression in an epigenetic manner. These results provide a theoretical base for breeding cucumber varieties with high flavonoid contents and provide a possible tool for discovering functional genes in cucumber.

## Supplementary data

Supplementary data are available at *JXB* online.

Fig. S1. Comparison of fruit skin colour between white-spined RNS8 and black-spined RNS9 at different developmental stages.

Fig. S2. Different metabolites between black and white spines at 2 DAA and 12 DAA.

Fig. S3. Preliminary chromatic test of the compounds in black spines using various chemical agents.

Fig. S4. Accumulation of flavonols in black spines at different developmental stages.

Fig. S5. NMR physicochemical and spectral data for the metabolites initially labelled as substances A–E.

Fig. S6. UV absorption spectrum, HPLC, and high-resolution MS data for the five metabolites A–E.

Fig. S7. SNP association at the *B* locus.

Fig. S8. Phylogenetic analysis of CsMYB60.

Fig. S9. Expression analysis of *CsMYB60* and key genes involved in flavonoid biosynthesis in cucumber spines.

Fig. S10. Additional biological repeats to determine the relative expression of *GUS* in transiently transformed cucumber cotyledons.

Fig. S11. Phylogenetic analysis of CsMudrA homologues in four species.

Fig. S12. DNA methylation status of the *CsMYB60* promoter in white-spined RNS8 and black-spined RNS9

Table S1. List of different metabolites between black spine and white spine.

Table S2. Throughput of RNA-Seq.

Table S3. Primers used for PCR reactions.

Table S4. Differentially expressed genes between white and black cucumber spines.

Supplementary Figures S1-S11 and Tables S1-S3

Supplementary Table S4
