# Supplementary material for: CsMYB60 is a key regulator of flavonols and proanthocyanidans that determine the colour of fruit spines in cucumber
Source: J Exp Bot. 2018 Sep 26;70(1):69–84. doi: 10.1093/jxb/ery336 (PMC6305189; doi:10.1093/jxb/ery336)
Supplement: Supplementary Figures S1-S11 and Tables S1-S3 [file ery336_suppl_supplementary_figures_s1-s12_tables_s1-s3.pdf]

***CsMYB60* is a key regulator of flavonols and proanthocyanidans that determine the colour of fruit spines in cucumber**

Mengyu Li, Cunjia Zhan, Lixin Dua, QianQian Luan, Jialin Li, Aigang Yang, Xiaoquan Qi and Zhonghai Ren

SUPPLEMENTARY DATA

A

B

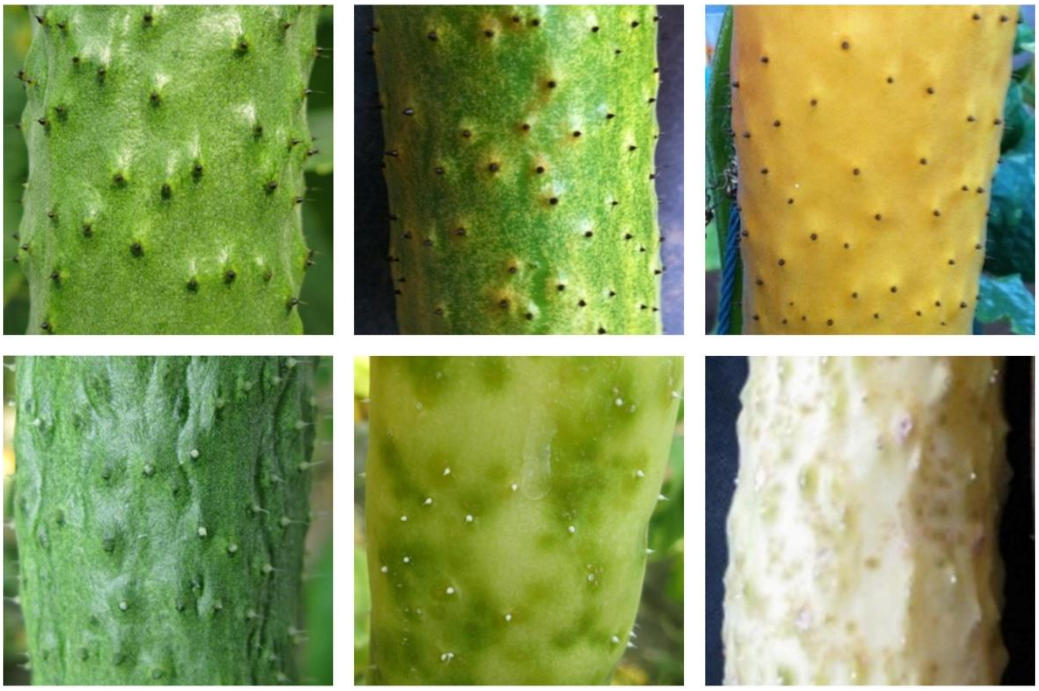

Figure S1. Comparison of fruit skin colour between white-spined RNS8 and black-spined RNS9 at different developmental stages. (A) The fruit of RNS9. From left to right, the development stages are 15DAA, 25DAA, and 40DAA. (B) The fruit of RNS8. From left to right, the development stages are 15DAA, 25DAA, and 40DAA.

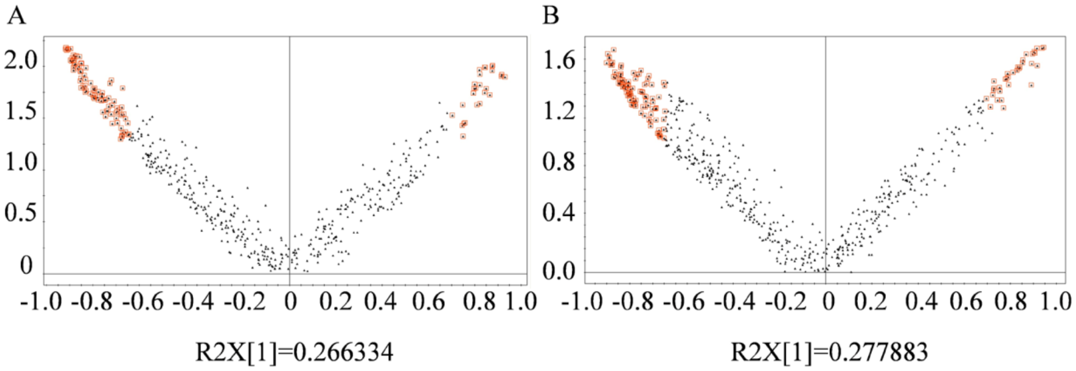

Figure S2. Different metabolites between black and white spines at 2DAA and 12DAA. (A) Different metabolites between black and white spines at 2DAA. (B) Different metabolites between black and white spines at 12DAA. Red boxes represent different metabolites. Ordinate values represent the Variable Importance for the Projection (VIP). Abscissa values represent coefficients (p(corr)). The screening criteria is VIP>1 and p (corr)>0.7 or <-0.7.

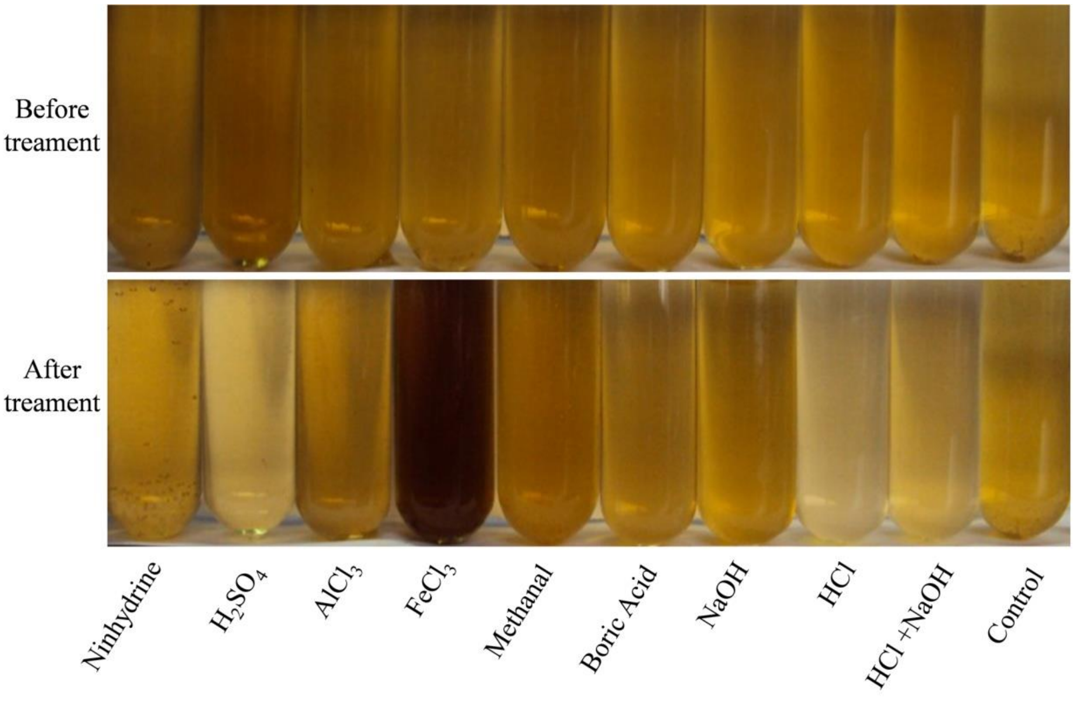

Figure S3. Preliminary chromatic test of the compounds in black spines using ninhydrine, H_2SO_4, $AlCl_3$, $FeCl_3$, methanal, boric acid, NaOH, HCl, and HCl + NaOH.

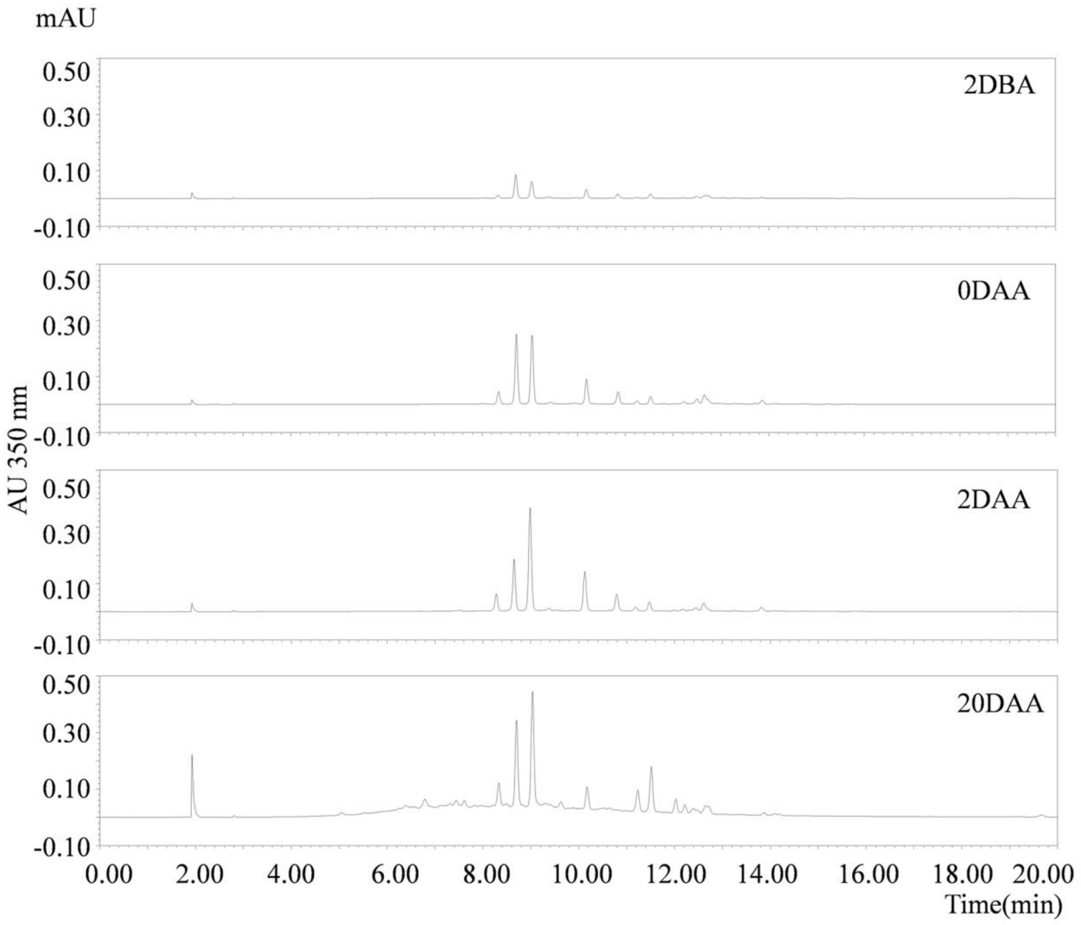

Figure S4. Accumulation of flavonols in black spines at different developmental stages. 2 DBA: 2 days before anthesis. 0 DAA: 0 days after anthesis. 2 DAA: 2 days after anthesis. 20 DAA: 20 days after anthesis.

Figure S5. The NMR Physicochemical and Spectral for the metabolites initially labelled as substances A–E.

Quercetin-3-O-rutinoside-7-O-glucose (Averett and Mabry, 1971**):** Light yellow powder, UV (MeOH), λ_{max} 213, 272, 337nm. ESI-MS m/z: 771.4,$[M-H]^-$. formular: $C_{33}H_{40}O_{21}$ ^{1}H-NMR (400 MHz, DMSO-d_6) δ: 7.52 (d, J=2.4 Hz, H-2'), 6.83 (d, J = 8.4 Hz, H-5'), 7.65 (dd, J=8.4, 2.4 Hz, H-6'), 6.39 (d, J = 2.0 Hz, H-8), 6.18 (d, J = 2.0 Hz, H-6), 5.46 (d, J=7.6 Hz, H-1''), 5.32 (d, J=7.6 Hz, H-1''''), 4.41 (brs, H-1'''), 0.95 (3H, d, J=6 Hz, Me-6'''); ^{13}C-NMR (100 MHz, DMSO-d_6) δ: 156.5(C-2), 133.4(C-3), 177.5(C-4), 161.4(C-5), 98.9(C-6), 164.6(C-7), 93.7(C-8), 156.2(C-9), 103.9(C-10), 121.3(C-1'), 116.1(C-2'), 148.7(C-3'), 145.0(C-4'), 115.3(C-5'), 122.1(C-6'), 101.0(C-1''), 74.2(C-2''), 76.6(C-3''), 70.8(C-4''), 76.0(C-5''), 65.2(C-6''), 100.1(C-1'''), 70.6(C-2'''), 70.1(C-3'''), 72.1(C-4'''), 68.2(C-5'''), 18.1(C-6'''), 100.1(C-1''''), 73.2(C-2''''), 76.6(C-3''''), 70.1(C-4''''), 77.7(C-5''''), 61.1(C-6'''').

Kempferol-3-O-rutinoside 7-O-glucoside (Budzianowski, 1990; Qu *et al.*, 2011**):** Light yellow powder, UV (MeOH), λ_{max} 217, 270, 340nm. ESI-MS m/z: 755.4 $[M-H]^-$, formular: $C_{33}H_{40}O_{20}$. ^{1}H-NMR (400 MHz, DMSO-d_6) *δ*: 6.18 (1H, d, *J* = 2.0 Hz, H-6),6.40 (1H, d, *J* = 2.0 Hz, H-8), 7.96 (2H, d, *J* = 8.5 Hz,H-2′, 6′), 6.88 (2H, d, *J* = 8.5 Hz, H-3′, 5′), 5.48 (1H,d, *J* = 7.2 Hz，H-1″)，5.03 (1H, d, *J* = 7.0 Hz，H-1''''),4.45 (1H, s, H-1‴), 1.02 (3H, d, *J* = 6.2 Hz, H-6‴). ^{13}C-NMR (100 MHz, DMSO-d_6)*δ*: 157.0 (C-2), 133.0(C-3), 177.6 (C-4), 159.9 (C-5), 98.2 (C-6), 162.8(C-7), 94.6 (C-8), 156.2 (C-9), 104.9 (C-10), 120.8(C-1′), 130.8 (C-2′, 6′), 159.3 (C-4′), 115.1 (C-3′, 5′),101.4 (C-1″), 74.5 (C-2″), 76.4 (C-3″), 70.5 (C-4″),75.2 (C-5″), 66.2 (C-6″), 100.7 (C-1‴), 70.7 (C-2‴),69.5 (C-3‴), 71.7 (C-4‴), 68.9 (C-5‴), 17.3 (C-6‴),100.0 (C-1''''), 73.5 (C-2''''), 76.1 (C-3''''), 69.0 (C-4''''),76.5 (C-5''''), 60.1 (C-6'''').

Isorhamnetin-3-O-rutinoside-7-O-glucoside (Parker and Bohm, 1975; Ito *et al.*, 2000**):** Light yellow powder, UV (MeOH), λ_{max} 213, 272, 337nm. ESI-MS m/z: 785.2, $[M-H]^-$, formular: $C_{34}H_{42}O_{21}$, ^{1}H NMR (DMSO-d_6): d 7.88 (1H, d, J=2 Hz, H-2'), 7.56 (1H, dd, J=2, 8 Hz, H-6'), 6.94 (1H, d, J=8 Hz, H-5'), 6.65 (1H, s, H-6), 5.43 (1H, d, J=8 Hz, H-1''), 5.06 (1H, d, J=7:5 Hz, H-1''''), 4.39 (1H, br s, H-1'''), 3.83 (3H, s, 3'-OMe), 0.95 (3H, d, J . 6 Hz, H-6'''). ^{13}C-NMR (100 MHz, DMSO-d_6) *δ*: 157.4 (C-2),

132.7 (C-3), 176.8(C-4), 160.5 (C-5), 99.2 (C-6), 161.9 (C-7), 94.9 (C-8),156.2 (C-9), 105.9 (C-10), 121.2 (C-1′), 113.3 (C-2′),147.1 (C-3′), 148.3 (C-4′), 115.6 (C-5′), 122.5 (C-6′), 55.7(C-3′-OMe),101.4 (C-1″), 74.5 (C-2″), 76.8(C-3″), 70.3 (C-4″),76.1 (C-5″), 67.2 (C-6″), 101.1 (C-1‴), 70.5 (C-2‴),70.8 (C-3‴), 71.9 (C-4‴), 68.4 (C-5‴), 17.9 (C-6‴),100.4(C-1⁗), 73.4 (C-2⁗), 77.4 (C-3⁗), 69.8 (C-4⁗),76.5 (C-5⁗), 61.5 (C-6⁗).

Kaempferol-3-O-rutinnoside (Markham *et al.*, 1978; Wang *et al.*, 2006**):** Light yellow powder, UV (MeOH), λ_{max} 214, 276, 342nm. ESI-MS m/z: 593.2, $[M-H]^-$, formular: $C_{27}H_{30}O_{15}$. ^{1}H-NMR (400 MHz, DMSO-d_6) δ: 12.57(1H ,5-OH), 6.20(1H , d , J =2.00 Hz , H-6), 6.40(1H ,d , J =2.00 Hz , H-8), 7.96(2H , dd , J =2.04 , 8.36Hz , H-2′, 6′), 6.87(2H, dd , J =2.04 , 8.36Hz , H-3′, 5′), 5.29(1H , d , J =7.80 Hz , H-1″), 4.42(1H, s, H-1‴), 0.89(3H , d , J =6.04 Hz , H-6‴); ^{13}C-NMR (100 MHz, DMSO-d_6) δ: 157.1(C-2), 133.3(C-3), 177.8(C-4), 160.6(C-5),98.7(C-6), 164.4(C-7), 93.8(C-8), 156.5(C-9), 103.8(C-10), 120.8(C-1′), 130.6(C-2′, 6′), 116.3(C-3′, 5′), 159.9(C-4′), 101.4(C-1″), 74.2(C-2″), 76.4(C-3″),70.3(C-4″), 76.0(C-5″), 66.5(C-6″), 100.6(C-1‴), 70.5(C-2‴), 70.4(C-3‴),71.6(C-4‴), 68.3(C-5‴), 18.0(C-6‴).

Isorhamnetin-3-O-rutinoside (Wen *et al.*, 2013**):** Light yellow powder, UV (MeOH), λ_{max} 218, 270, 340nm. ESI-MS m/z: 623.2, $[M-H]^-$, formular: $C_{28}H_{32}O_{16}$, ^{1}H-NMR(400 MHz, DMSO-d_6) δ: DMSO-*d6*) δ: 12.56 (s, 5-OH), 7.85 (d, J = 2.4 Hz, H-2′), 6.90 (d, J = 8.4 Hz, H-5′), 7.51 (dd, J = 8.4, 2.4Hz, H-6′), 6.42 (d, J = 2.0 Hz, H-8)、6.18 (d, J = 2.0Hz, H-6), 3.83 (3H, s, 3′-OMe), 5.43 (d, J = 7.2 Hz, H-1″), 5.38 (d, J = 3.2 Hz, H-1‴), 0.97 (3H,d, J = 6.0 Hz, H-6‴); ^{13}C-NMR(100 MHz, DMSO-d_6) δ: 156.3 (C-2), 132.7 (C-3), 176.8(C-4), 160.5 (C-5), 98.7 (C-6), 164.3 (C-7), 93.5 (C-8),156.2 (C-9), 103.9 (C-10), 120.9 (C-1′), 115.0 (C-2′),149.2 (C-3′), 146.7 (C-4′), 113.1 (C-5′), 122.1 (C-6′), 55.5(C-3′-OMe) ,101.0 (C-1″), 74.4 (C-2″), 76.4 (C-3″), 70.1(C-4″), 75.8 (C-5″), 66.7 (C-6″), 100.1(C-1‴), 70.6 (C-2‴), 70.5 (C-3‴), 71.9(C-4‴), 68.7 (C-5‴), 17.5 (C-6‴).

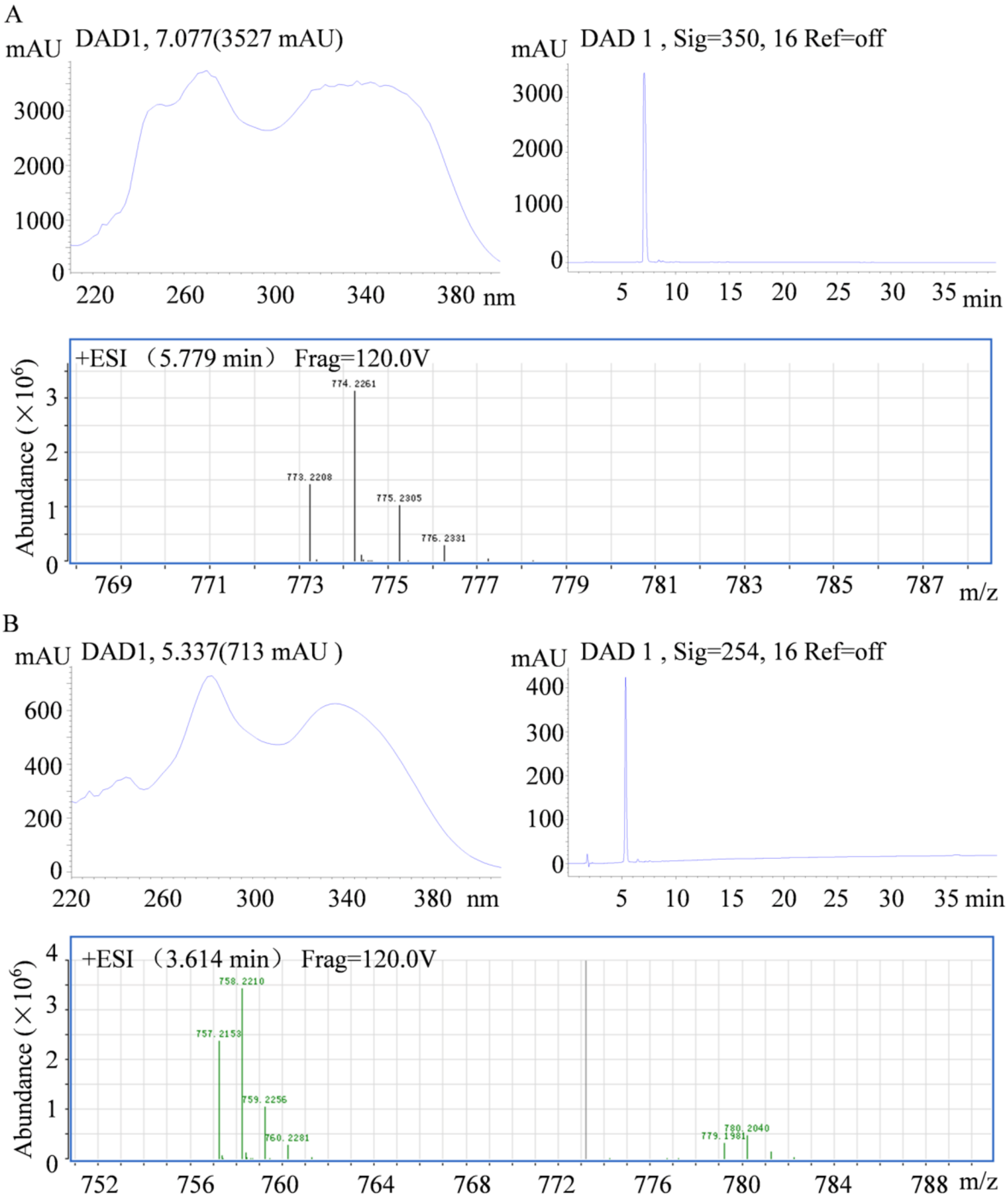
A
mAU DAD1, 7.077(3527 mAU)
mAU DAD 1 , Sig=350, 16 Ref=off
+ESI (5.779 min) Frag=120.0V
774.2261
773.2208
775.2305
776.2331
Abundance (×10^6)
m/z
nm
min
B
mAU DAD1, 5.337(713 mAU)
mAU DAD 1 , Sig=254, 16 Ref=off
+ESI (3.614 min) Frag=120.0V
758.2210
757.2153
759.2256
760.2281
779.1981
780.2040
Abundance (×10^6)
m/z

C

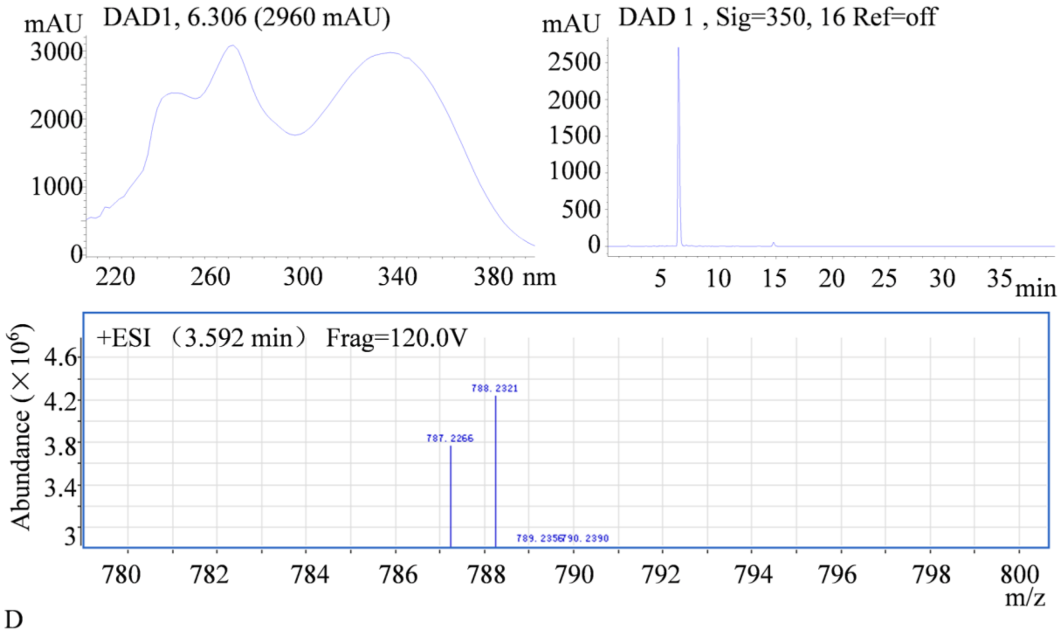
mAU
DAD1, 6.306 (2960 mAU)
3000
2000
1000
0
220
260
300
340
380 nm
mAU
DAD 1 , Sig=350, 16 Ref=off
2500
2000
1500
1000
500
0
5
10
15
20
25
30
35
min
+ESI （3.592 min） Frag=120.0V
Abundance (×10^6)
4.6
4.2
3.8
3.4
3
787.2266
788.2321
789.2356
790.2390
780
782
784
786
788
790
792
794
796
798
800
m/z

D

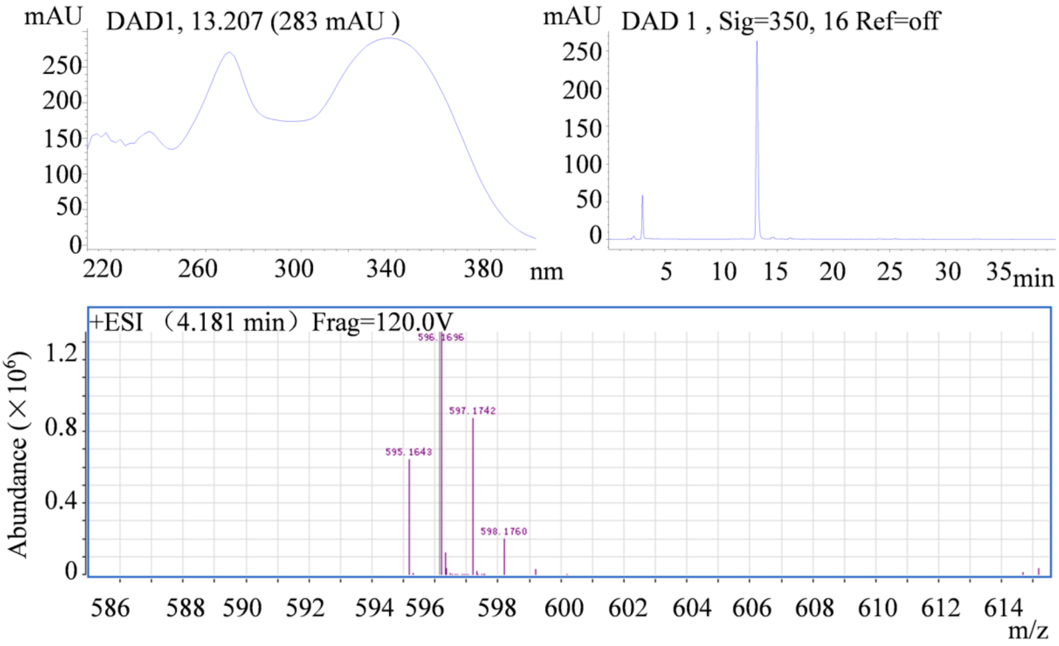
mAU
DAD1, 13.207 (283 mAU)
250
200
150
100
50
0
220
260
300
340
380
nm
mAU
DAD 1 , Sig=350, 16 Ref=off
250
200
150
100
50
0
5
10
15
20
25
30
35
min
+ESI （4.181 min）Frag=120.0V
Abundance (×10^6)
1.2
0.8
0.4
0
595.1643
596.1696
597.1742
598.1760
586
588
590
592
594
596
598
600
602
604
606
608
610
612
614
m/z

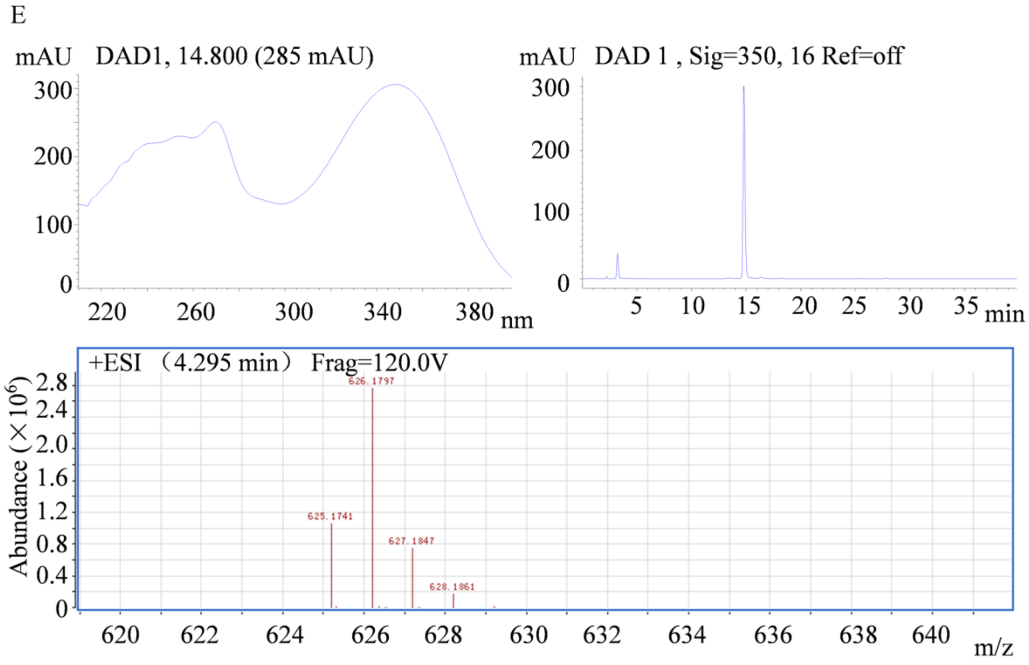

Figure S6. Ultraviolet absorption spectrum, high-performance liquid chromatography and high-resolution mass spectrometry data for the five metabolites A–E**.** (A) A: $C_{33}H_{40}O_{21}$. (B) B: $C_{33}H_{40}O_{20}$. (C) C: $C_{34}H_{42}O_{21}$. (D) D: $C_{27}H_{30}O_{15}$. (E) E: $C_{28}H_{32}O_{16}$.

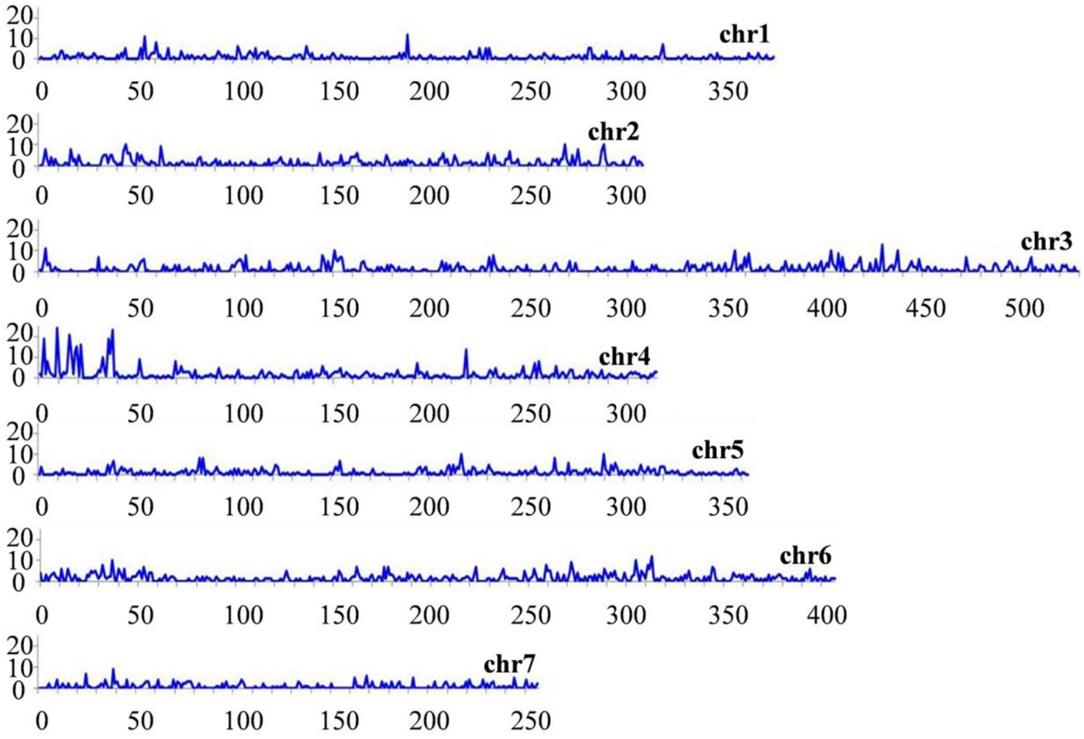

Figure S7. SNP association at the *B* locus. White and black spine groups from the F2 population derived from a cross between RNS8 and RNS9 were used for RNA-seq, and the resultant SNPs were used for association analysis.

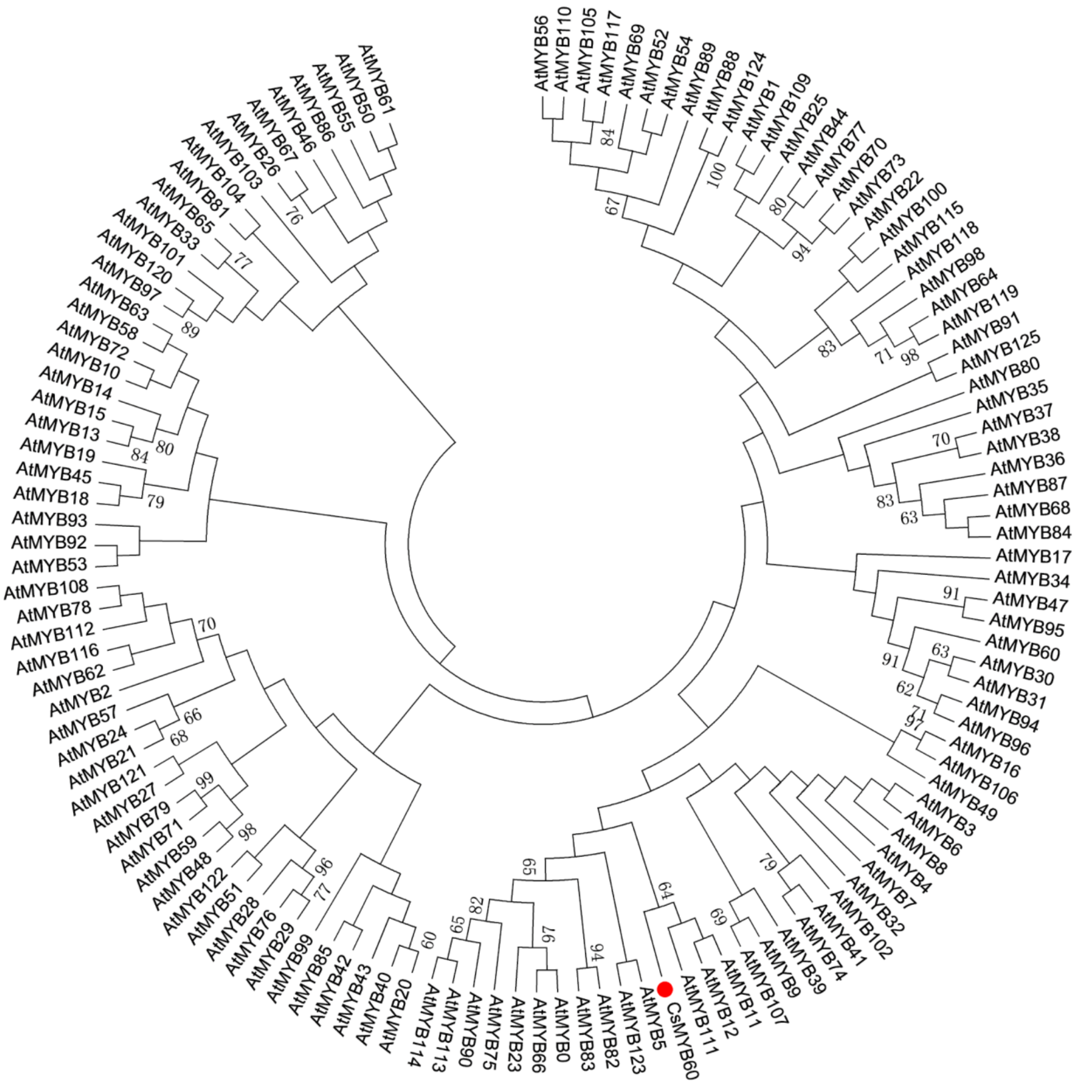

Fig S8. Phylogenetic analysis of CsMYB60. The neighbor-joining tree includes CsMYB60 and 126 R2R3MYB proteins from Arabidopsis. CsMYB60 is highlighted with red circle. The phylogenetic tree was constructed with MEGA5 using a bootstrap test of phylogeny with UPGMA test and default parameters. The numbers above or below branches indicate bootstrap values (>50%) from 1000 replicates.

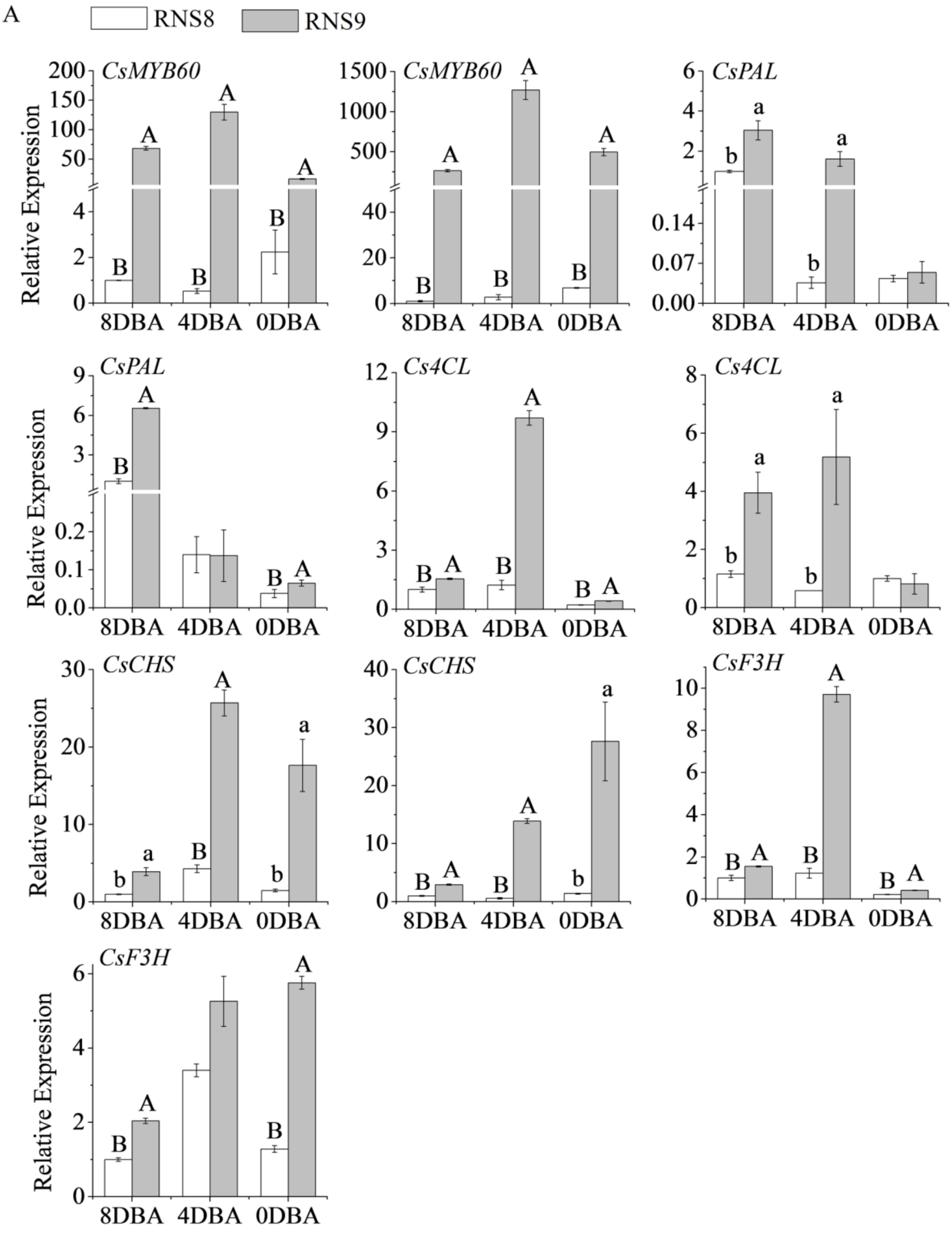
A
RNS8
RNS9
CsMYB60
CsMYB60
CsPAL
CsPAL
Cs4CL
Cs4CL
CsCHS
CsCHS
CsF3H
CsF3H
Relative Expression
8DBA
4DBA
0DBA

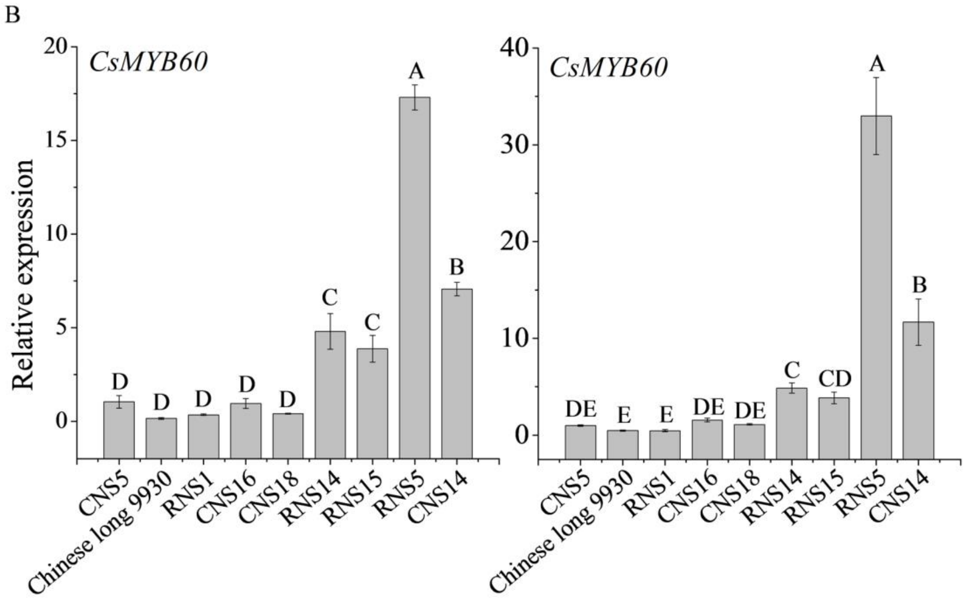

Figure S9. Expression analysis of *CsMYB60* and key genes involved in flavonoid biosynthesis in cucumber spine. (A) The other two biological repeats to determine the expression of key genes in spines at different developmental stages. qPCR was used to assess gene transcript accumulation in total RNA samples extracted from 8DBA, 4DBA, and 0DBA fruit spines. Capital and small letters indicate significant differences at q=0.01 and q=0.05, respectively. (B) The expression of *CsMYB60* in fruit spines from different cucumber inbred lines. qPCR was used to assess *CsMYB60* transcript accumulation in total RNA samples extracted from fruit spine at 0DAA from different cucumber inbred lines. Different letters indicate significant differences at q=0.01.

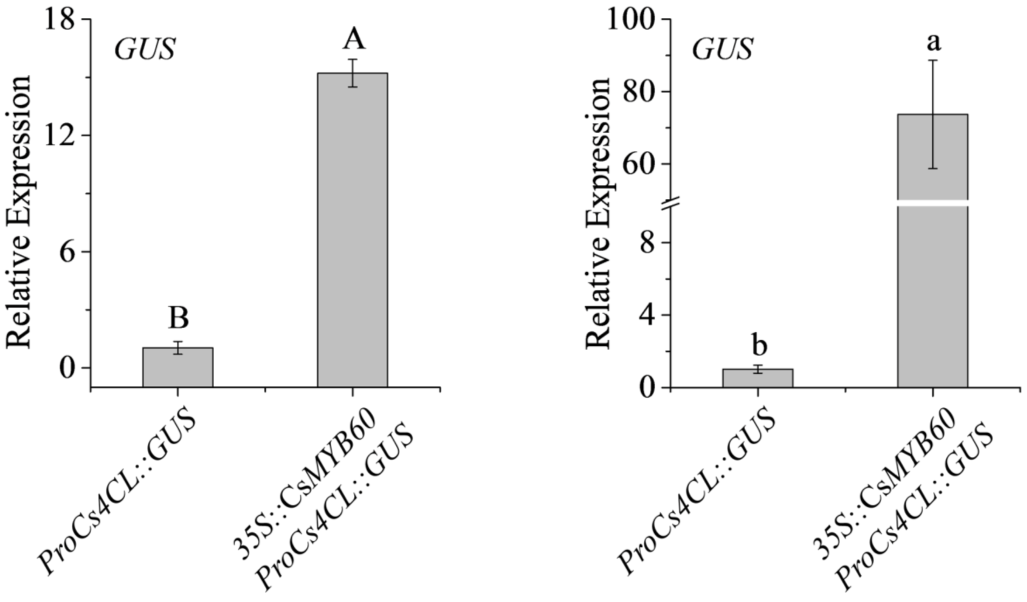

Figure S10. Additional biological repeats to determine the relative expression of *GUS* in transiently transformed cucumber cotyledons. Capital and small letters indicate significant differences at q=0.01 and q=0.05, respectively.

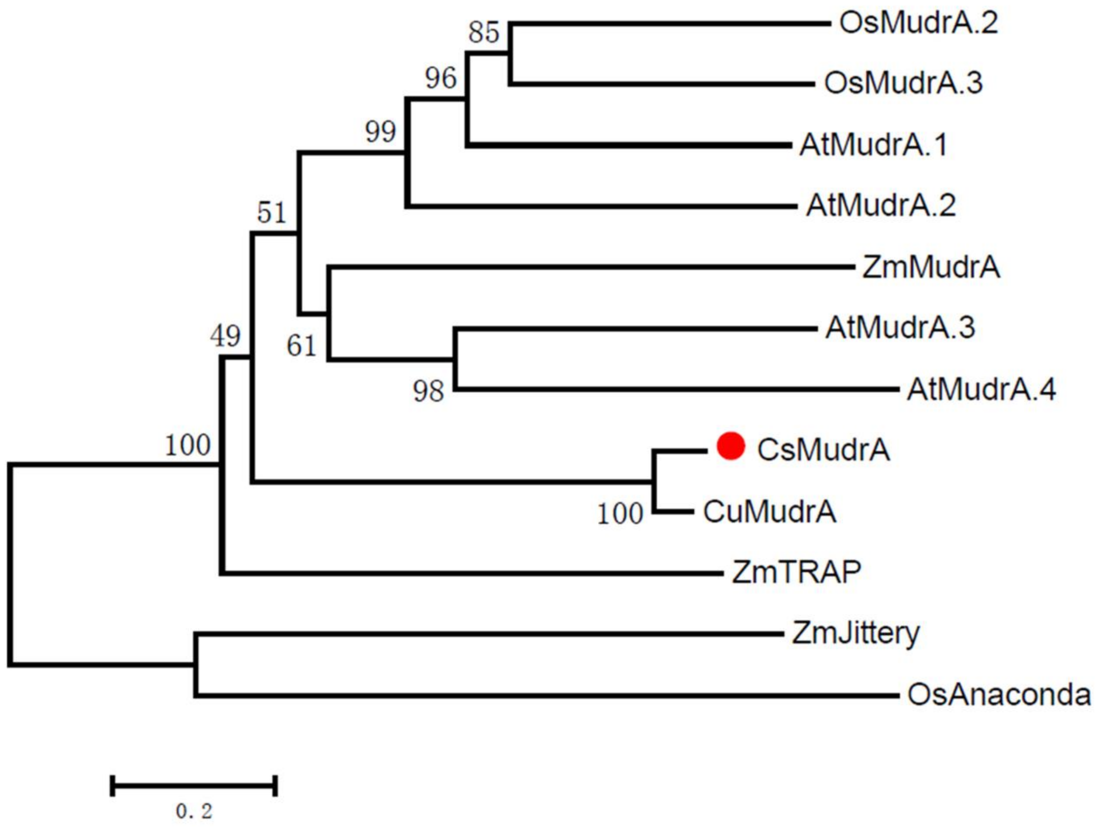

Figure S11. Phylogenetic analysis of CsMudrA homologues in four species. The phylogenetic tree was constructed by the neighbour-joining method with 1,000 bootstrap replicates using the software MEGA5.2. CsMudrA (AUS83695), ZmMudrA (AAA21566.1), CuMudrA (AAU04773), AtMudrA.1 (AAF26998.1), AtMudrA.2 (AAF63144.1), AtMudrA.3 (AAG51216.1), AtMudrA.4 (AAD50007.1), OsMudrA.2 (AP000366), OsMudrA.3 (AAK00423), OsMudrA.7 (AB023047), ZmJittery (AF247646.1), ZmTRAP (CAB51950.1), OsAnaconda (BAD26733).

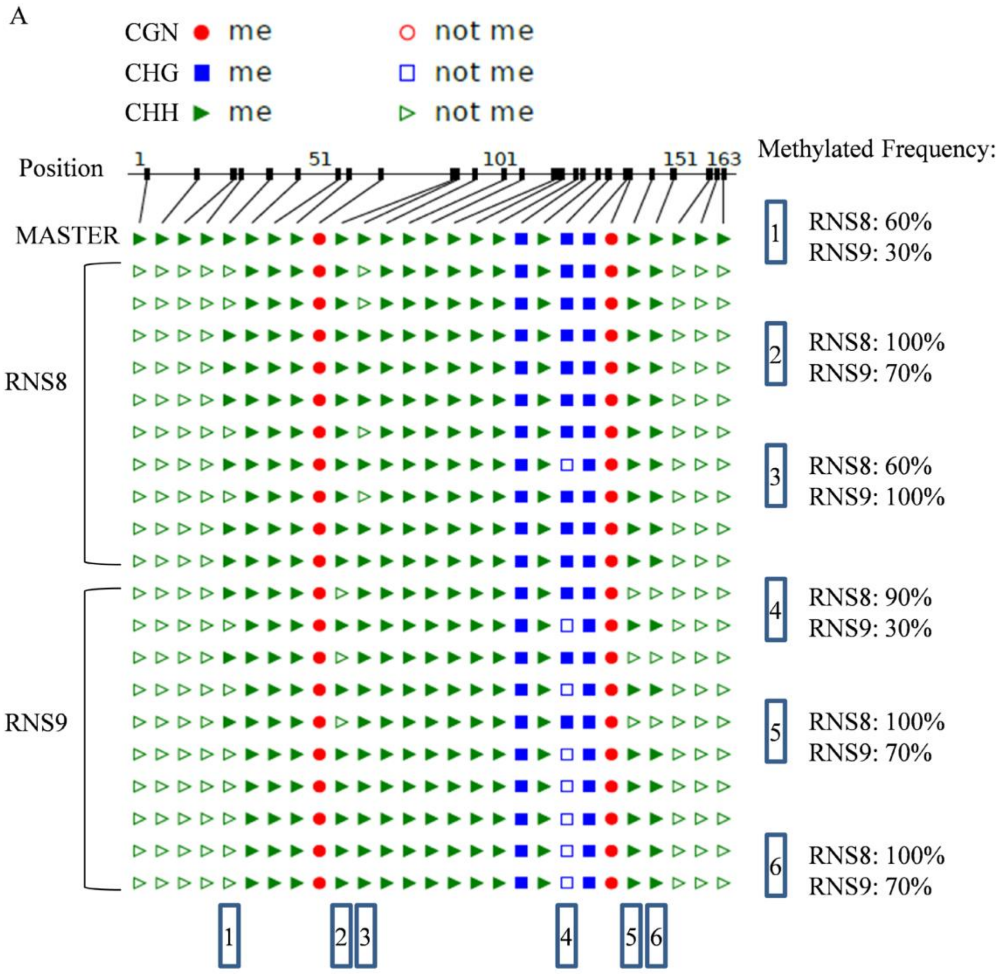
A
CGN me
not me
CHG me
not me
CHH me
not me
Position
1
51
101
151
163
MASTER
RNS8
RNS9
Methylated Frequency:
1 RNS8: 60%
RNS9: 30%
2 RNS8: 100%
RNS9: 70%
3 RNS8: 60%
RNS9: 100%
4 RNS8: 90%
RNS9: 30%
5 RNS8: 100%
RNS9: 70%
6 RNS8: 100%
RNS9: 70%
1
2
3
4
5
6

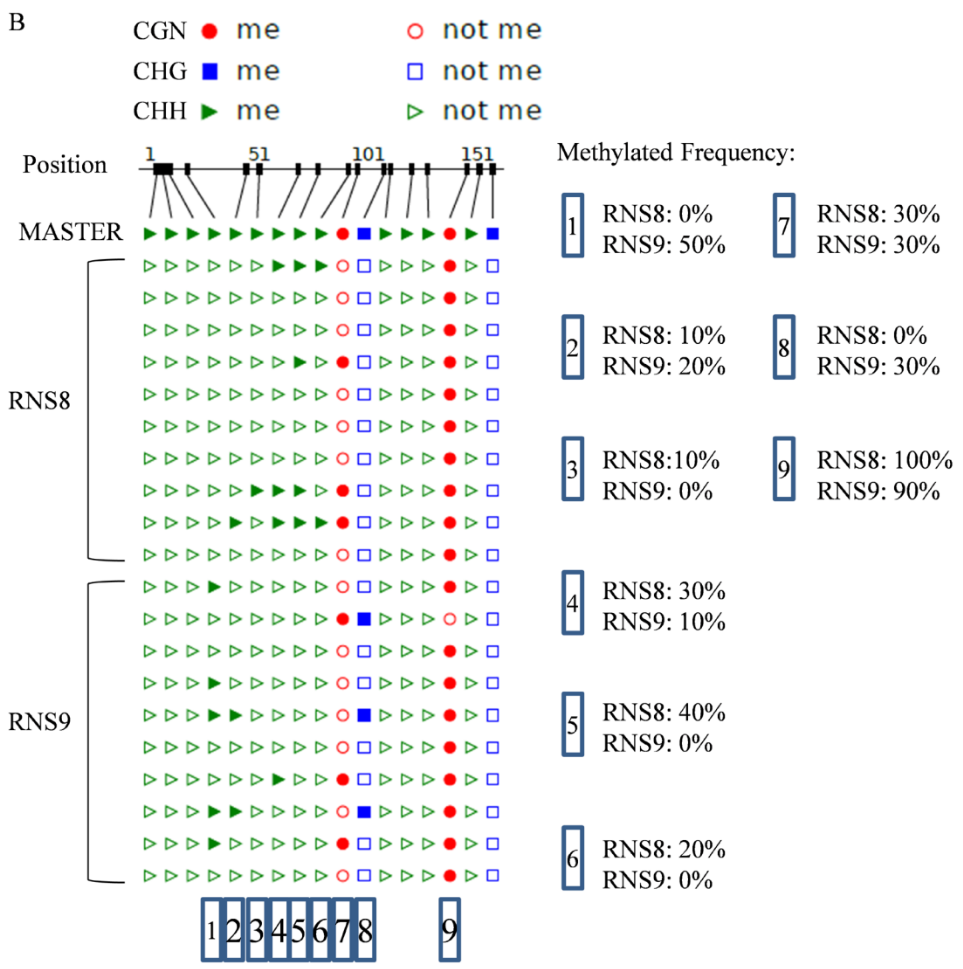

Fig S12. DNA methylation status of the *CsMYB60* promoter in white-spined RNS8 and black-spined RNS9. DNA methylation status of the promoter region at -4701 ~ -4514 (A) and at -3855 ~ -3692 (B) before the ATG of *CsMYB60* in RNS8 and RNS9. CyMATE generates images in which filled symbols represent cytosine methylation, while open symbols represent lack of methylation. The sequence context is distinguished by red circles for mCG, blue squares for mCHG and green triangles for mCHH. Numbers along the top show the length of the alignment.

Table S1. List of different metabolites between black spine and white spines, represented by the *m*/*z* ratio at the retention time.

2DAA	12DAA
276.2097@20.46193	398.3012@25.953728
630.1362@20.929388	726.4531@23.002153
423.3564@21.456627	365.3664@32.359375
398.3012@25.953728	330.2777@26.130033
363.3355@24.63563	328.0586@2.9477143
406.1835@0.87950003	644.4637@31.425533
365.3664@32.359375	505.3035@12.721153
255.2564@18.307486	448.1015@2.9386315
184.0586@0.56246877	642.2876@30.019318
242.1766@0.643826	432.1597@0.95737034
725.5228@33.091316	348.0828@1.465871
448.1015@2.9386315	479.4197@27.192999
636.2995@27.133514	756.2119@0.96800005
725.5238@33.563457	452.3376@12.717455
430.3779@28.847744	270.0079@4.5144854
432.1055@3.9819374	317.2335@21.695435
369.2881@23.721344	594.1588@0.97014284
414.2051@19.138916	970.5775@29.123001
263.2249@14.941636	724.3976@27.131472
794.186@0.96220005	371.1922@25.71919
798.5275@33.01426	559.379@30.216597
466.1454@2.2998095	669.4625@30.709164
479.1408@2.87715	393.3454@25.955215
423.3689@25.0232	753.5547@33.098606
309.3036@32.064	525.3665@13.085576
479.4197@27.192999	749.5447@28.21154
226.1688@1.3733715	602.4374@28.107466
317.2335@21.695435	585.4172@12.178799
594.1588@0.97014284	474.1501@16.988317
517.293@24.599049	516.3638@24.154408
601.426@25.940868	399.3118@28.355097
371.1922@25.71919	411.3122@28.592415
853.3062@0.559	290.1882@20.789616
370.0941@27.482521	423.2732@12.464642
669.4625@30.709164	676.2907@26.814833

749.5447@28.21154	331.3095@18.590708
474.1501@16.988317	398.1706@13.873478
399.3118@28.355097	408.3722@27.602257
411.3122@28.592415	514.1086@11.232097
710.487@31.87085	375.2057@17.642277
676.2907@26.814833	775.5652@23.48724
331.3095@18.590708	414.2042@18.405518
408.3722@27.602257	547.348@13.085242
747.5342@20.95924	717.5243@22.3265
463.4236@29.794134	954.562@30.693941
375.2057@17.642277	703.5085@21.017366
775.5652@23.48724	492.3475@20.708458
717.5243@22.3265	462.1165@1.0107999
443.3239@22.124155	576.3858@21.2004
561.3184@24.489128	307.2516@25.248465
545.3631@29.646957	548.0482@6.003176
307.2514@25.107574	307.2514@25.107574
697.9801@11.469866	611.4983@29.37587
740.3444@21.943619	745.5548@24.899467
743.5766@29.04291	628.468@32.522503
595.503@31.954365	718.4208@33.57063
859.6604@31.437416	596.4083@12.181243
579.5077@32.06768	950.1197@2.9444287
544.1918@7.0803127	740.3444@21.943619
268.2017@21.575787	743.4035@18.21896
422.3874@28.346727	359.3202@29.531563
429.2996@25.767586	348.1765@1.260111
697.4782@11.468454	685.5341@27.855764
743.4032@18.357578	579.5077@32.06768
342.3246@23.375374	678.3568@30.01017
380.1088@1.29955	641.3754@26.751968
576.1968@8.919818	544.1918@7.0803127
279.2565@20.572657	268.2017@21.575787
626.4531@32.893482	521.2456@2.0056248
683.5551@31.752697	290.2465@21.536907
585.4452@22.592545	690.3903@31.869884
651.4715@29.917004	512.1503@1.6769501

307.2723@21.538002	743.4032@18.357578
821.6013@33.007355	340.3092@22.026443
598.1089@22.45513	576.1968@8.919818
304.1497@1.0248519	626.4531@32.893482
613.4765@25.27374	683.5551@31.752697
294.2201@22.30163	576.9134@13.27525
583.4663@26.77866	598.1089@22.45513
668.4637@30.560095	632.1353@1.6414735
289.2621@17.575531	727.582@31.66572
643.1433@4.9128127	278.2251@25.452322
653.3251@27.1336	445.4135@25.198036
612.4763@29.86337	375.3119@25.169466
477.2865@20.053839	668.4637@30.560095
103.1001@0.63449997	352.0775@0.99924004
305.2571@11.5143585	380.145@2.1256666
634.4582@33.63487	643.1433@4.9128127
759.578@26.341267	375.3124@28.235136
571.43@21.199572	477.2865@20.053839
243.0256@2.94675	305.2571@11.5143585
210.0661@2.007889	634.4582@33.63487
285.3032@22.221169	395.3375@22.19403
317.2335@22.544165	321.2674@25.730852
492.1272@10.2553005	367.3068@19.737404
471.3108@12.716342	285.3034@19.777172
271.2512@17.8442	778.1932@0.96823066
317.2331@21.861158	357.2647@27.659376
452.3268@33.88443	918.2437@8.472
550.3485@23.40397	624.1698@1.2973334
312.2283@21.536627	218.1673@14.942709
255.2564@17.451374	202.1318@0.7055626
457.341@23.587116	271.2512@17.8442
356.2549@21.50263	333.2881@15.226371
407.302@28.847343	317.2331@21.861158
609.1769@30.307144	452.3268@33.88443
395.3256@21.456114	302.0432@5.9805765
439.3518@21.395517	109.0017@0.56326467
359.1819@21.943739	932.2592@10.480258

587.4036@13.276515	702.4469@33.569057
283.3247@24.258629	255.2564@17.451374
665.3627@30.001284	415.3488@22.94391
405.3466@17.6208	330.2773@26.589487
335.2844@27.654226	765.5238@22.911312
819.5869@31.532751	317.2337@22.015888
	457.341@23.587116
	356.2549@21.50263
	430.3782@29.224573
	407.302@28.847343
	609.1769@30.307144
	348.0826@1.7033157
	331.0601@4.8982997
	181.0059@4.514001
	262.0219@4.5150886
	404.1661@2.0072072
	358.227@11.472943
	482.1406@1.4592692
	261.2307@10.5396
	587.4036@13.276515
	383.3025@17.500656
	283.3247@24.258629
	610.1544@2.3019524
	301.2991@19.589973
	700.4871@11.476572
	273.2679@17.266771

Number@Number (e.g. 276.2097@20.46193) means mass-to-charge ratio (*m*/*z*) at retention time.

Table S2. Throughput of RNA-Seq.

Sampling time	Samples	Total reads number	Total mapped reads	Mapped	GC percentage
2 DAA	RNS8-1	52269372	49250425	94.22%	43.32%
	RNS8-2	56434978	53304601	94.45%	43.42%
	RNS8-3	53951280	51415304	95.3%	43.41%
	RNS9-1	57677546	54172735	93.92%	43.30%
	RNS9-2	70940836	66737681	94.08%	43.39%
	RNS9-3	48002920	44930420	93.60%	43.32%

Table S3. Primers used for PCR reactions.

Name	Sequence（5'-3'）
CsMYB60-F	ATGGAGAGAGAGAGTGGTGGG
CsMYB60-R	TCCGATTCCGATTCCGCAAC
Cs4CL-F	ACGGATTGACAGAGACCG
Cs4CL-R	GAAACTCCGTCTCTTTGTACTC
CsPAL-F	AACTTGAAGAGCACTGTG
CsPAL-R	GGTGAAGTGTCCCATTAGAGG
CsCHS-F	GCTTAAAGATGTACCTGGCC
CsCHS-R	CAACACACACGCGCTTGAC
CsF3H-F	ATGACTTTTTCTCTCCCACATC
CsF3H-R	CTCCGTCGGAAGACCAAAGAAC
GUS-F	ATGTTACGTCCTGTAGAAACCCCA
GUS-R	ACATACGGCGTGACATCGGCTTC
Actin-F	ATGGCCGATGCCGAGGATATT
Actin-R	CTTTTCTCTGTTAGCCTTTGGG
ProCs4CL-F	AAGCTTCAATTTCAACCCTAAAACCC
ProCs4CL-R	GGATCCAAAATGGGAATGGGATG
CsMYB60-GUS-F	GGATCCATGGGGAGAAGGCCGTGC
CsMYB60-GUS-R	GAGCTCTCATGACAAAAGCCAAGAAATC
CsMYB60-956-F	ACAACCGACAGCATCCTT
CsMYB60-956-R	GGTATGTAACGAACCTGC
CsMYB60-2010-F	TTTAGCCCTCCCACCATA
CsMYB60-2010-R	GTGCTTAATGGCATGTGG
CsMYB60-3236-F	GACACACCATCAATAGGG
CsMYB60-3236-R	CCATCGTACACGTCCATT
CsMYB60-4869-F	CTAATTCGTCAACCCCTC
CsMYB60-4869-R	CAGACCAATCACAGCCTA
CsMYB60-5695-F	AAATCCGGTCATTCCCCA

CsMYB60-5695-R	CGGAACTGTTGTCAGTGA
CsMYB60-6362-F	GCCACGAAAACCTACACA
CsMYB60-6362-R	ACGCCCAATTTCACAGAG
CsMYB60-6002-F	GAATCGGGTAGATGGTGTTG
CsMYB60-6002-R	GCCCTTTGAGTGTAATCAATCC
CsMYB60-5469-F	GGATTGATTACACTCAAAGGGC
CsMYB60-5469-R	GCAGAGACTTCTTGGAATAG
CsMYB60-5124-F	CCCAAGGTTAGCACAACATC
CsMYB60-5124-R	GGACCCTATTGTCCTTTTG
CsMYB60-4567-F	CAAAAGGACAATAGGGTCC
CsMYB60-4567-R	GTTGTCAGTGATAGAGTC
CsMYB60-4182-F	CACACACTTGGTGATATAG
CsMYB60-4182-F	CAATCCAAACGTCCTCTTAG
CsMYB60-4008-F	GCACAACATGAACATAGG
CsMYB60-4008-R	TTGAAGAGCACTCAATAC
CsMYB60-3674-F	CTAAGAGGACGTTTGGATTG
CsMYB60-3674-R	GCATCTAACCATTGCTTG
CsMYB60-3221-F	CTGCTAATTGTTTCAATA
CsMYB60-3221-R	CCCTATTGATGGTGTGTC
CsMYB60-3006-F	CAAGCAATGGTTAGATGC
CsMYB60-3006-R	CAGACCAATCACAGCCTA
